# Cohort-based pan-cancer analysis and experimental studies reveal ISG15 gene as a novel biomarker for prognosis and immunotherapy efficacy prediction

**DOI:** 10.1007/s00262-025-04026-y

**Published:** 2025-04-10

**Authors:** Jingjing Wei, Yingjia Zhuang, Chengfei Jiang, Lingyan Chen, Binbin Yuan, Yue Zhao, Happi Li, Jian-Hua Mao, Bo Hang, Chunping Ye, Lei Wang, Pin Wang

**Affiliations:** 1 Department of Gastroenterology, Nanjing Drum Tower Hospital Clinical College of Nanjing University of Chinese Medicine, Nanjing, Jiangsu China; 2 Department of Gastroenterology, Nanjing Drum Tower Hospital, Clinical College of Nanjing Medical University, Nanjing, Jiangsu China; 3 Department of Ultrasound, Kunshan Hospital of Traditional Chinese Medicine, Kunshan, Jiangsu China; 4 Department of Gastroenterology, Nanjing Drum Tower Hospital, Affiliated Hospital of Medical School, Nanjing University, Nanjing, Jiangsu China; 5 Department of Gynecology, The First Affiliated Hospital of Nanjing Medical University, Nanjing, Jiangsu China; 6 Saratoga High School, 20300 Herriman Ave, Saratoga, CA USA; 7 Biological Systems and Engineering Division, Lawrence Berkeley National Laboratory, Berkeley, CA USA; 8 Department of Obstetrics and Gynecology, Nanjing Maternity and Child Health Care Hospital, Women’s Hospital of Nanjing Medical University, Nanjing, Jiangsu China; 9 Department of Liver Surgery and Transplantation, Liver Cancer Institute, Zhongshan Hospital, Fudan University, Shanghai, China

**Keywords:** Interferon-stimulated gene ISG15, Pan-cancer, Tumor microenvironment, Prediction of immunotherapy efficacy, Immune checkpoint inhibitors, Prognostic biomarkers

## Abstract

ISG15, an interferon-stimulated ubiquitin-like protein, plays a multifaceted role in tumorigenesis and immune regulation. This study comprehensively evaluates ISG15 as a prognostic biomarker and predictor of immunotherapy response through pan-cancer bioinformatics analysis and experimental validation. By integrating multiomics data from TCGA, GEO, and clinical cohorts, we found that ISG15 is significantly overexpressed in multiple cancers and generally correlates with poor prognosis. Elevated ISG15 expression is associated with increased immune checkpoint gene expression, particularly PD-L1, and immune infiltration, notably M2-like tumor-associated macrophages. Immunohistochemistry and multiplexed immunofluorescence confirmed a strong positive correlation between ISG15, PD-L1, and M2-TAM infiltration in lung and gastric cancer samples. Functional analysis at the single-cell level revealed significant associations between ISG15 and tumor proliferation, angiogenesis, and immune suppression. Immunotherapy cohort analysis demonstrated that tumors with high ISG15 expression responded favorably to PD-L1 inhibitors but exhibited resistance to CTLA-4 blockade, findings further validated in lung cancer patients receiving anti-PD-1 therapy. These results suggest that ISG15 is a promising biomarker for prognosis and immunotherapy response prediction across cancers. Its integration into clinical decision-making may enhance personalized treatment strategies, improve immunotherapy outcomes, and provide new insights into the tumor immune microenvironment, cancer progression, and potential therapeutic targets for future drug development.

**Supplementary Information:**

The online version contains supplementary material available at 10.1007/s00262-025-04026-y.

## Introduction

ISG15 is one of the earliest discovered ubiquitin-like proteins [1, 2]. It can be robustly upregulated by Type I interferon and covalently binds to lysine residues in substrate proteins, a process known as ISGylation [3]. The enzymatic cascade reaction of ISG15 covalent modification is divided into two stages: ISGylation refers to the catalyzation of the covalent binding of ISG15’s di-glycine sequence to target proteins through ubiquitin-activating enzyme E1 (UB31L), ubiquitin-conjugating enzyme E2, and ubiquitin ligase enzyme E3; de-ISGylation refers to the dissociation of ISG15 from target proteins by ubiquitin-specific peptidase (USP18) [4, 5]. ISG15 is considered a key regulatory factor in maintaining protein homeostasis and participates in the regulation of cancer and immune diseases [6, 7]. Up to now, in vivo studies on ISGylation in tumor occurrence are relatively scarce, thus understanding the role of ISG15 in tumor development is of great importance.

Host inflammation, immunity, and susceptibility represent a complex response driven by various factors [8]. The collaborative cross (CC) mouse model has emerged in recent years as a novel research tool with genetic and phenotypic diversities closely paralleling humans, accompanied by a wide range of spontaneous tumor developments [9]. For instance, after screening 30 CC strains, our previous study identified spontaneous gastric cancer in the CC036 strain for the first time. The gastric tissues of CC036 exhibited an overexpression of 15 inflammation-associated genes including *ISG15* early on, in comparison with those resistant CC strains, which are regulated by NFκB1. Subsequent investigations revealed that gastric cancer patients with elevated *ISG15* expression demonstrate a markedly decreased overall survival (OS) rate [10]. Concurrently, we observed that CC036 mice presented heightened levels of lymphocytes and neutrophils, with an augmented presence of both B and T cell subsets. Therefore, we postulate that *ISG15* plays an instrumental role in the progression of gastric cancer in CC036 mice by involving inflammation and immune dysregulation. Furthermore, ISG15 has been implicated in multiple tumorigenic processes beyond immune dysregulation. Elevated ISG15 expression has been shown to inhibit the activity of cytotoxic T lymphocytes and natural killer cells, thereby facilitating tumor growth and invasion [11]. Additionally, ISG15 is associated with tumor angiogenesis, as its inhibition in certain cancers reduces the secretion of angiogenesis-related factors such as VEGF and IL-6, leading to suppressed tumor growth and invasiveness [12]. ISG15 also interacts with immune checkpoint proteins like PD-L1 through ISGylation, a ubiquitin-like modification process [13]. This interaction enhances PD-L1 ubiquitination, promoting its degradation via the ubiquitin–proteasome pathway. These mechanisms highlight ISG15’s pivotal role in shaping the tumor microenvironment and underscore its potential as a therapeutic target.

With the rapid development and integration of oncology, immunology, and molecular biology, our understanding of the tumor microenvironment and various immune checkpoints has significantly deepened [14, 15]. Within the microenvironment, T cells exhibit high expression of PD-1, while tumor cells prominently express PD-L1, resulting in the continuous activation of the PD-1/PD-L1 pathway, inhibiting T cell’s ability to recognize tumor cells, and compromising the immune system’s attack on tumor cells [16]. Moreover, tumor cells can induce the differentiation of various infiltrating immune cells into tolerogenic immune cells, such as tumor-induced regulatory T cells (Tregs) and tumor-associated macrophages (TAMs), which lose their immunosurveillance capacity, leading to immune evasion [17, 18]. Immune therapy has become a vital component of comprehensive cancer treatment. Developments are primarily seen in adoptive cell therapies, exemplified by chimeric antigen receptor T cells (CAR-T), and targeted treatments against negative immune checkpoints, such as the PD-1/PD-L1 targeted therapy. A great effort has been made toward the development of novel and combination antitumor immunotherapies targeting various mechanisms [19–22]. In ICI (immune checkpoint inhibitors) therapy, anti-PD-1 and anti-PD-L1 antibodies, as well as anti-CTLA-4 antibodies, have proved highly effective for patients with microsatellite instability-high (MSI-H) subtype or elevated PD-L1 expression [19, 23, 24]. The application of tumor immune therapy still faces numerous challenges, such as the susceptibility of only a small fraction of tumors, overall low clinical efficacy, and difficulties in accurately predicting therapeutic outcomes and responses. Overcoming these limitations needs the identification of new immunotherapeutic biomarkers [25]. Current biomarkers, such as PD-L1 and tumor mutation burden (TMB), often suffer from limited applicability and inconsistent predictive outcomes across tumor types. For instance, PD-L1 expression does not always correlate with response to PD-1/PD-L1 inhibitors, and TMB is often associated with high costs and technical challenges in clinical testing. ISG15, through its role in immune infiltration and modulation, may address these limitations by offering a broader and more precise predictive capability for immunotherapy responses across diverse cancers.

In this study, we employed numerous pan-cancer databases for the first time to perform a pan-cancer analysis on *ISG15* together with validations. Based on comprehensive multiomics analyses of *ISG15*, encompassing gene expression, prognostic analysis, immune infiltration, immune relevance, gene mutation analysis, single-cell level tumor functional status analysis, and immune therapeutic response analysis, we systematically explored its pathogenic mechanism, clinical prognostic value, and predictive value for immunotherapy outcome in various tumors. Simultaneously, we applied our clinical cohort samples to validate the aforementioned results. The findings of this study may provide comprehensive information about ISG15 in predicting tumor patient prognosis and the responses to immunotherapy. The integration of ISG15 into clinical practice could provide valuable insights for patient stratification and therapy selection. For example, assessing ISG15 expression levels in tumor biopsies may help identify patients more likely to benefit from immune checkpoint inhibitors or combination therapies, paving the way for more personalized treatment strategies.

## Materials and methods

### Gene and protein expression analysis

We analyzed ISG15 mRNA expression across various tumor and normal tissues using TCGA-PanCancer data from UCSC Xena. TIMER2 was used to compare ISG15 expression in tumor and adjacent normal tissues, and GTEx data from GEPIA2 were used to supplement TIMER2 where normal tissue data were unavailable. Protein-level expression differences were assessed using CPTAC data from UALCAN and immunohistochemical images from the Human Protein Atlas (HPA). Detailed methods and database access information are provided in Supplementary Methods 1.

### Gene mutation and copy number variation analysis

We analyzed ISG15 gene mutations and their proportions across pan-cancer using data from cBioPortal. Kaplan–Meier survival curves were generated to evaluate the impact of ISG15 mutations on overall survival, progression-free survival, and disease-free survival (DFS). Additionally, copy number variation (CNV) data from TCGA-PanCancer were analyzed to assess the frequency of ISG15 CNVs in different cancers and their correlation with mRNA expression levels. Detailed statistical methods and data processing steps are provided in Supplementary Methods 2.

### Single-cell level tumor functional status analysis

Single-cell sequencing data for ISG15 expression were analyzed using CancerSEA, a database comprising 72 single-cell datasets across 25 cancer types. We evaluated ISG15 expression and its correlation with 14 tumor functional states, highlighting significant correlations in acute myeloid leukemia (AML), retinoblastoma (RB), and uveal melanoma (UM). t-SNE plots were utilized to visualize ISG15 expression distribution. Detailed methods and analysis steps are provided in Supplementary Methods 3.

### Survival prognosis analysis

To evaluate the prognostic impact of ISG15 expression, we utilized the “Survival Analysis-Survival Map” module on GEPIA2 to identify tumors where OS and DFS were significantly influenced by ISG15 expression. Tumors with significant differences were further analyzed using Kaplan–Meier survival curves with a 50% cutoff to distinguish high and low expression groups. Additionally, meta-gene chip data for eight tumors were analyzed using Kaplan–Meier plotter to validate the findings, and survival analysis on intrahepatic cholangiocarcinoma patients from Zhongshan Hospital was conducted with a follow-up period exceeding five years. Detailed methods and statistical parameters are provided in Supplementary Methods 4.

### Establishment and evaluation of the nomogram models

Firstly, a univariate and multivariate Cox regression analysis was conducted on tumors to assess whether ISG15 expression affects OS prognosis and to determine if ISG15 expression serves as an independent risk factor. Subsequently, from the analyzed tumors, those with a significant *p*-value (*p* < 0.05) and a substantial sample size were chosen to individually construct nomogram models. These models offer an efficient and user-friendly method for predicting OS in individual patients. Calibration curves were utilized to evaluate the predictive accuracy of the nomograms at the 3-year and 5-year intervals. For kidney renal clear cell carcinoma (KIRC) patients, the maximum follow-up time was 149 months, and for SKCM patients, it exceeded 369 months.

### Immune infiltration analysis

We explored the relationship between ISG15 expression and immune infiltration across cancers using TIMER2 and CIBERSORT algorithms. Cancer-associated fibroblast infiltration was analyzed using Spearman’s rank correlation, and immune cell infiltration differences between high and low ISG15 expression groups were compared. Additionally, we assessed the correlation between ISG15 and 24 immune checkpoint markers across cancers and performed cluster analysis of ISG15 expression across immune subtypes. Detailed methods and statistical analyses are provided in Supplementary Methods 5.

### Relationship between ISG15 and immunotherapy outcomes

We analyzed the impact of ISG15 expression on immunotherapy outcomes in patients treated with anti-PD-1, anti-PD-L1, and anti-CTLA-4 therapies using Kaplan–Meier plotter and IMvigor210 datasets. Survival curves illustrated the influence of ISG15 expression on OS in nine cancer types, including bladder cancer, glioblastoma, and hepatocellular carcinoma. Additionally, immunohistochemical analysis was performed on lung cancer tissues to evaluate ISG15 expression levels in relation to treatment response to anti-PD-1 therapy. Patients were categorized into high and low ISG15 expression groups based on Immunohistochemistry scores, and treatment efficacy was compared using chi-square tests. Detailed methods and statistical parameters are provided in Supplementary Methods 6.

### Immunohistochemistry (IHC) and multiplexed immunofluorescence (IF) staining

Tissue samples from 12 lung cancer and 10 gastric cancer patients were analyzed using IHC and multiplexed IF staining to assess ISG15 and PD-L1 expression, as well as macrophage infiltration (CD68 and CD206 markers). Sections were prepared and stained using standard protocols, and quantitative analyses were performed using the PerkinElmer Vectra Polaris Automated Quantitative Pathology Imaging System. Detailed experimental procedures and antibody information are provided in Supplementary Methods 7.

### Macrophage polarization assay and flow cytometry analysis

The human gastric cancer cell line AGS (Shanghai Institute of Biochemistry and Cell Biology, Shanghai, China) was transduced with lentiviruses to overexpress ISG15 and USP18 (Corues, Nanjing, China) and selected with puromycin (2 μg/mL) for three days. Overexpression efficiency was confirmed by qPCR. THP-1 monocytes (Shanghai Institute of Biochemistry and Cell Biology, Shanghai, China) were seeded at 800,000 cells per well in a six-well plate and differentiated into M0 macrophages by treating them with 100 ng/mL PMA (GlpBio, USA) for 24 h, followed by a 48-h resting period in fresh media. M0 macrophages were then co-cultured for 48 h with conditioned media collected from AGS-NC, AGS-OEISG15, and AGS-OEUSP18 cells. After co-culture, macrophages were detached using trypsin, washed three times with PBS, and stained for 40 min at 4 °C with PE antihuman CD86 (M1 marker, BD Pharmingen, #560957, USA, 1:100) and APC antihuman CD206 (M2 marker, BD Pharmingen, #550889, USA, 1:100). Cells were then washed three times with PBS, and 10,000 events were collected on a BD LSRFortessa™ flow cytometer. Flow cytometry data were analyzed using FlowJo v10 software, and the M2:M1 macrophage ratio was calculated based on the proportion of CD206⁺ (M2) and CD86⁺ (M1) macrophages. Statistical significance was determined using one-way ANOVA followed by Tukey’s post hoc test, with data presented as mean ± SEM and *p* < 0.05 considered statistically significant.

### Data processing and statistical analysis

Tumor data from TCGA were normalized using the FPKM method, and data from the IMvigor210 dataset were normalized using the CPM method with the edgeR package. Statistical analyses included the Wilcoxon rank-sum test, Spearman correlation, chi-square test, Kaplan–Meier survival analysis, and Cox proportional hazards regression. Covariates included in Cox regression models for KIRC and SKCM were age, gender, tumor stage, and ISG15 expression. A nomogram prognostic model was constructed based on these analyses. All statistical analyses were performed using R (version 4.0.2) and SPSS (version 26.0), with *p*-values < 0.05 considered statistically significant. Detailed data processing steps and statistical parameters are provided in Supplementary Methods 8.

## Results

### The expression alteration of ISG15 in pan-cancer

**Fig. 1:**
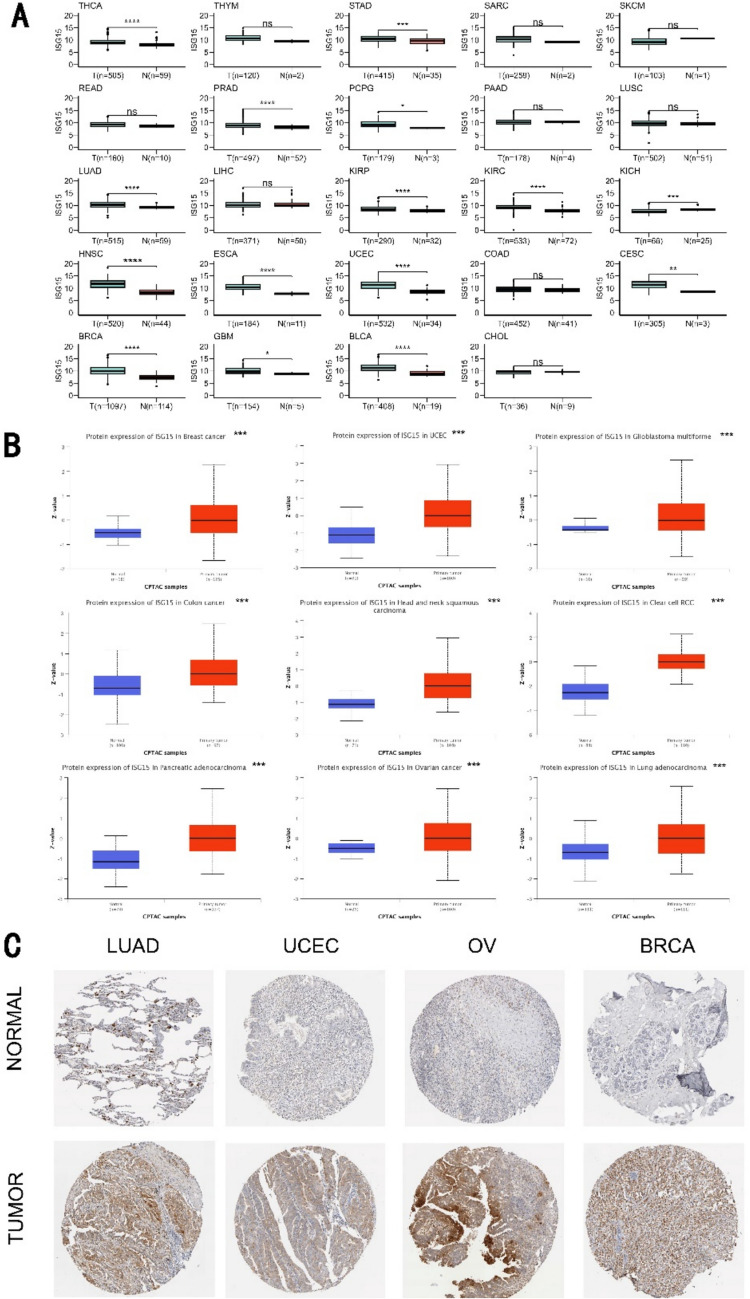
Differential Expression of ISG15 in Tumoral and Normal Tissues. **A** Evaluation of ISG15 mRNA expression levels within neoplastic and normative specimens, utilizing the TCGA-PanCancer database. **B** Comparative analysis of total ISG15 protein between tumoral and regular tissue, sourced from the CPTAC dataset via the UALCAN web portal. **C** Immunohistochemistry (IHC) visualizations, obtained from the Human Protein Atlas (HPA) platform, employed to validate the disparate expression of ISG15 protein. *P < 0.05; **P < 0.01; ***P < 0.001

Given the reported involvement of ISG15 in various cancer types, we first assessed the alteration of ISG15 in pan-cancers. Figure 1A shows that ISG15 is significantly overexpressed in THCA (thyroid carcinoma), STAD (stomach adenocarcinoma), PRAD (prostate adenocarcinoma), PCPG (pheochromocytoma and paraganglioma), LUAD (lung adenocarcinoma), KIRP (kidney renal papillary cell carcinoma), KIRC, HNSC (head and neck squamous cell carcinoma), ESCA (esophageal carcinoma), UCEC (uterine corpus endometrial carcinoma), CESC (Cervical squamous cell carcinoma), BRCA (breast invasive carcinoma), GBM (glioblastoma multiforme), and BLCA (bladder urothelial carcinoma), while it is underexpressed only in KICH (kidney chromophobe). These findings were further confirmed by comparing the expression of *ISG15* mRNA in pan-cancer tumor tissues and corresponding normal tissues within the TIMER2 and GTEx databases (see Supplementary Fig. S1A, B). Moreover, we analyzed the correlation between ISG15 expression levels and pathological stages in several tumors via GEPIA2 (see Supplementary Fig. S1C). ISG15 expression is significantly associated with different pathological stages in several cancers, including KIRC, PAAD (pancreatic adenocarcinoma), and SKCM (skin cutaneous melanoma), suggesting a potential role of ISG15 expression levels in dictating pathological progression.

We also evaluated the expression of ISG15 protein in tumors using the CPTAC dataset. In contrast to normal tissues, ISG15 was found to be markedly expressed in breast cancer, uterine corpus endometrial carcinoma, glioblastoma multiforme, colon cancer, head and neck squamous carcinoma, clear cell renal cell carcinoma, pancreatic adenocarcinoma, ovarian cancer, and lung adenocarcinoma (Fig. 1B). Moreover, we explored ISG15 protein expression in normal and tumor tissues from diverse human organs utilizing the HPA, and similar results were observed. Figure 1C demonstrates representative IHC images of normal and tumor tissues of the lung, ovary, uterus, and breast. Taking together, we conclude that both mRNA and protein levels of ISG15 are significantly elevated in most cancer types examined in this study.

**Fig. 2:**
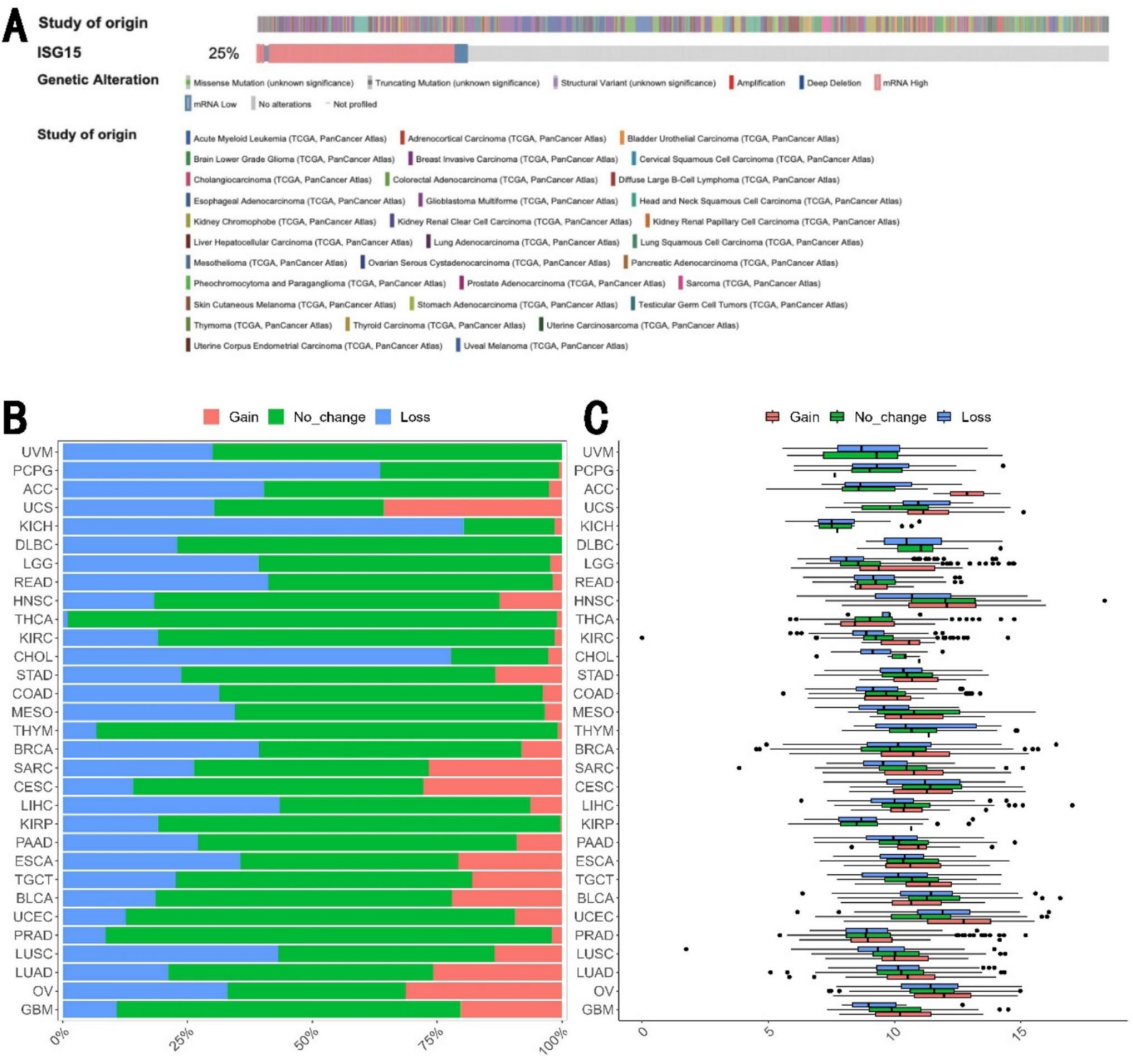
Mutation of ISG15 in Pan-Cancer. **A** The genetic alteration of ISG15 across various tumors utilizing the TCGA database via the cBioPortal website. **B** Incidence of genomic alterations in ISG15 across various human cancer modalities. **C** Interplay between ISG15 mRNA expression levels and genomic alterations

To explore the possible mechanisms for ISG15 overexpression in human cancer, we examined the genetic alteration of *ISG15* across various tumors utilizing the TCGA database via the cBioPortal website. Mutation of *ISG15* was found only in 17 of 10,967 cancers. 25% of cancers have alteration in *ISG15* where majority of cases have overexpression of ISG15 (Fig. 2A), consistent with elevation of *ISG15* expression in human cancer. Surprisingly, we found that ISG15 was lost in over 20% of cases across 21 out of the 31 human cancer types, while ISG15 was gained in over 20% of cases across 8 of 31 cancer types (Fig. 2B). Notably, *ISG15* expression is significantly higher in transcriptional expression in tumors exhibiting a gain of ISG15, relative to those without change in some cancer types, suggesting that the gain of ISG15 orchestrates the subsequent elevation in ISG15 expression (Fig. 2C, Supplementary Table S1). In the context of BRCA, a gain in ISG15 was associated with a notable elevation in ISG15 expression (Fig. 2C, Supplementary Table S1). Upon surveying the available data for LGG (brain lower grade glioma), HNSC, KIRC, SARC (sarcoma), LIHC (liver hepatocellular carcinoma), UCEC, and LUSC (lung squamous cell carcinoma), the expression of ISG15 exhibited a positive correlation with ISG15 loss (Supplementary Table S1). Intriguingly, in the instance of UCEC, ISG15 was expressed at significantly elevated levels in cases with an *ISG15* loss (Fig. 2C, Supplementary Table S1). These findings suggest that additional mechanisms might propel the increased expression of *ISG15*.

### Prognostic value of ISG15 across pan-cancer

**Fig. 3:**
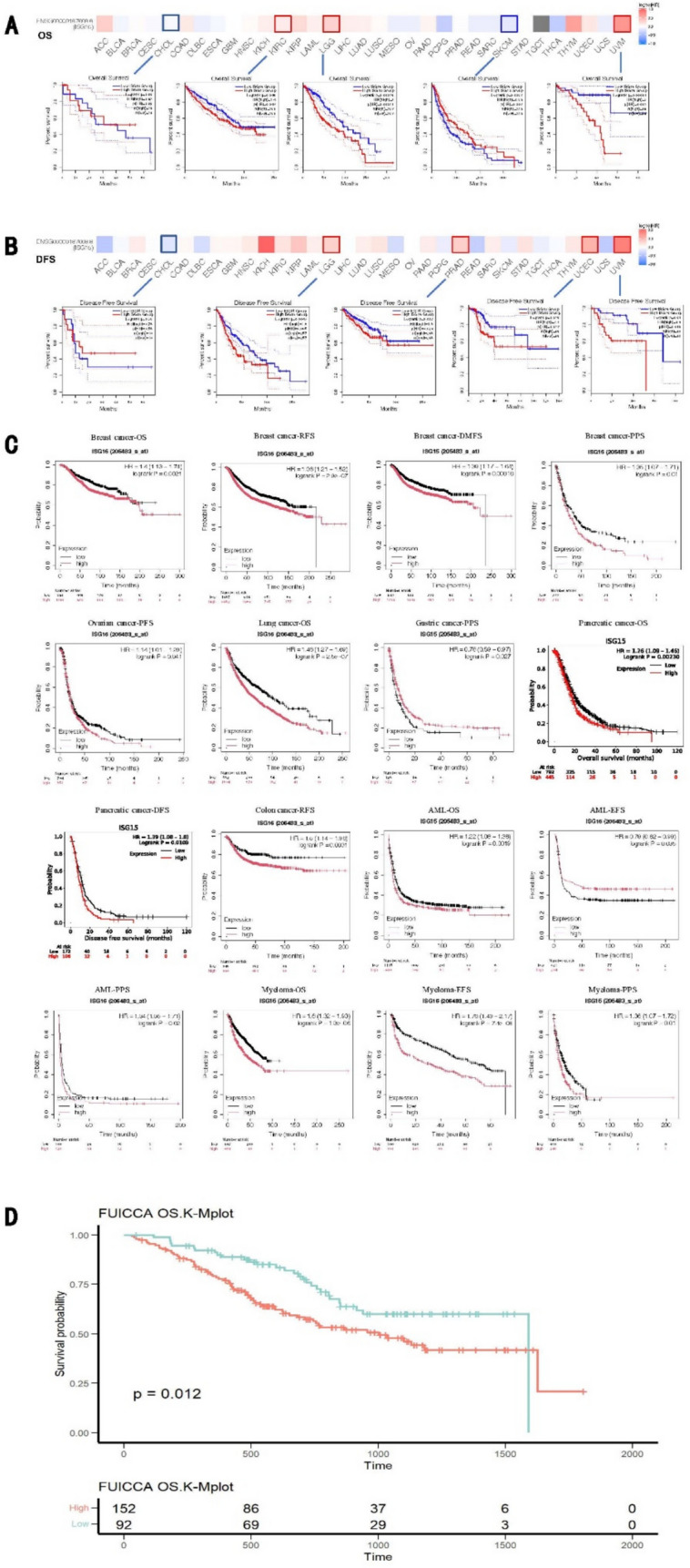
Interplay between ISG15 Expression Levels and Pan-Cancer Prognostic Survival. The implicational role of ISG15 expression levels on OS **A** and DFS **B** within tumors is delineated utilizing the GEPIA2 database, which incorporates survival curves for CHOL, KIRC, LGG, SKCM, UVM, PRAD, and UCEC. **C** A scrutiny of ISG15 gene expression’s influence on tumor prognosis, extracted from gene chip data available on the Kaplan–Meier plotter website. **D** Examination of the repercussion of ISG15 differential expression on the overall survival of cholangiocarcinoma patients, informed by data from Zhongshan Hospital. OS, overall survival; RFS, relapse-free survival; DFS, disease-free survival; DMFS, distant metastasis-free survival; DSS, disease-specific survival; PFS, progression-free survival; PPS, post-progression survival; EFS, event-free survival

We comprehensively assessed the prognostic significance of *ISG15* across pan-cancer. Based on our results from the GEPIA2 database, ISG15 expression levels notably correlated with the prognosis of KIRC, LGG, SKCM, UVM (uveal melanoma), PRAD, and UCEC (Fig. 3A, B). Elevated expression of ISG15 correlated with worse prognoses of these five tumors, KIRC (OS: total number = 516, Hazard Ratio, HR (high) = 1.4, log-rank *P* = 0.046), LGG (OS: total number = 514, HR (high) = 2, Log-rank *P* = 0.00016; DFS: total number = 514, HR (high) = 1.6, Log-rank *P* = 0.0047), PRAD (DFS: total number = 492, HR (high) = 1.6, log-rank *P* = 0.027), UCEC (DFS: total number = 172, HR (high) = 2.1, log-rank *P* = 0.025), and UVM (OS: total number = 78, HR (high) = 5, log-rank *P* = 0.00064; DFS: total number = 78, HR (high) = 3.4, log-rank *P* = 0.01). Conversely, diminished ISG15 expression led to unfavorable prognoses for SKCM tumors (OS: total number = 458, HR (high) = 0.69, log-rank *P* = 0.0067).

Next, we validated the prognostic value of ISG15 using the Kaplan–Meier plotter, which encompasses meta-microarray transcriptional data and clinical outcomes across many human cancer types. Consistent with the above findings, we found that for breast cancer, lung cancer, pancreatic cancer, AML, and myeloma, the expression levels of ISG15 markedly influence patients’ OS (breast cancer: OS, HR = 1.4, log-rank *P* = 0.0021; lung cancer: OS, HR = 1.46, log-rank *P* = 2.5e−07; pancreatic cancer: OS, HR = 1.26, log-rank *P* = 0.0023; AML: OS, HR = 1.22, log-rank *P* = 0.0019; myeloma: OS, HR = 1.6, log-rank P = 1.3e−06). Pertaining to breast cancer and colon cancer, ISG15 expression levels correlate with RFS (breast cancer: RFS, HR = 1.36, log-rank *P* = 2.9e−07; colon cancer: RFS, HR = 1.5, log-rank *P* = 0.0031). Lastly, the expression of ISG15 affects DMFS in breast cancer, PFS in ovarian cancer, DFS in pancreatic cancer, PPS in breast cancer, gastric cancer, AML, and myeloma, as well as EFS in AML and myeloma (Fig. 3C). Given the limited sample size of intrahepatic cholangiocarcinoma in TCGA, we utilized cohort data from 244 patients with intrahepatic cholangiocarcinoma from the Zhongshan Hospital for prognostic analysis and validated a significant association of high ISG15 expression levels with poor prognosis in patients (*p* = 0.012) (Fig. 3D).

**Fig. 4:**
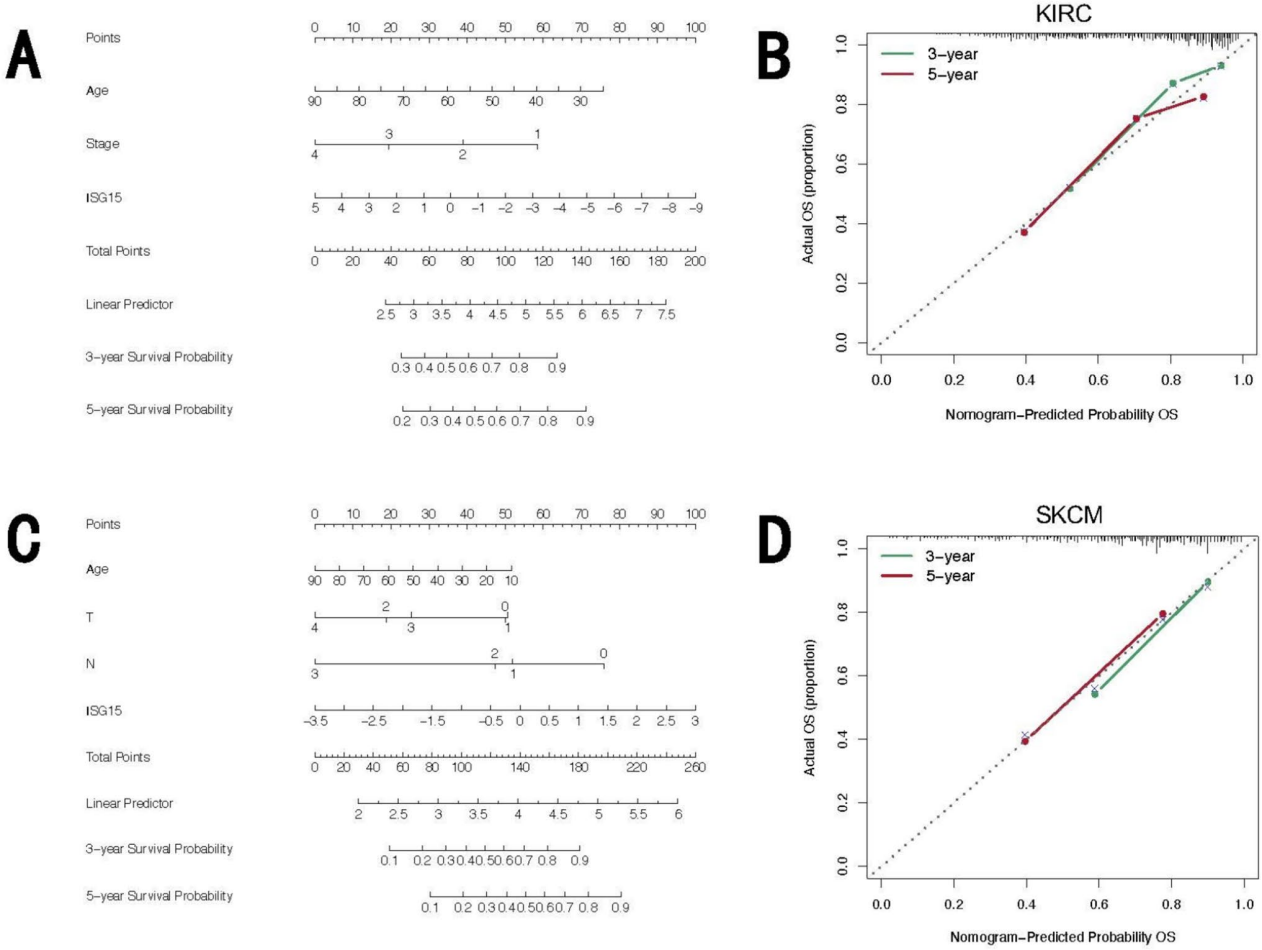
Significant prognostic value of ISG15 in KIRC and SKCM. Prognostic nomogram using the ISG15 expression and the clinical features to predict 3- and 5-year survival in patients with KIRC (**A**) and SKCM (**C**). The calibration curves were established to evaluate the predictive value of the nomogram models for the prognosis of KIRC (**B**) and SKCM (**D**) patients at 3 and 5 years

Finally, to elucidate whether the prognostic impact of ISG15 expression is independent of clinical factors, we conducted univariate and multivariate Cox regression analyses focusing on OS across five types of cancer wherein ISG15 has a demonstrable effect on prognostic outcomes (see Supplementary Tables S2–S6). Utilizing results from multivariate Cox regression analyses, KIRC and SKCM were chosen for the development of a nomogram model to independently assess prognostic value. Calibration curves were employed to assess the predictive precision of the nomogram model at 3-year and 5-year intervals. Our findings demonstrate that within these nomogram models, ISG15 plays a significant role in prediction and offers robust predictive capabilities for the OS of KIRC (Fig. 4A) and SKCM (Fig. 4C). Furthermore, the calibration curves for 3- and 5-year survival predictions underscore the high accuracy of the nomogram models in forecasting OS (Fig. 4B, D).

Taking together, we conclude that there is a significant correlation between ISG15 expression and prognosis, and in most cancer types, higher ISG15 expression is associated with poor prognosis.

### The impact of ISG15 expression levels on the functional status of tumor cells at a single-cell level

Single-cell sequencing technology can overcome issues related to tumor cell heterogeneity. Our correlation analysis of ISG15 expression with various tumor functional statuses, performed on the CancerSEA website, revealed substantial correlations with most tumor functional statuses (see Supplementary Fig. S2A). Detailed correlation coefficients are provided in Supplementary Table S7. We selected six significantly correlated tumor functional statuses (see Supplementary Fig. S2B), with AML’s differentiation, inflammation, proliferation, and metastasis, and RB’s angiogenesis and differentiation displaying notable positive correlations with ISG15 expression, while UM’s DNA repair showed a noticeable negative correlation. We also detailed the single-cell level expression profile of ISG15 in AML, RB, and UM (see Supplementary Fig. S2C). All these findings suggest that ISG15 participates in the biological progression of cancer.

### Correlation of ISG15 expression with tumor immunity

**Fig. 5:**
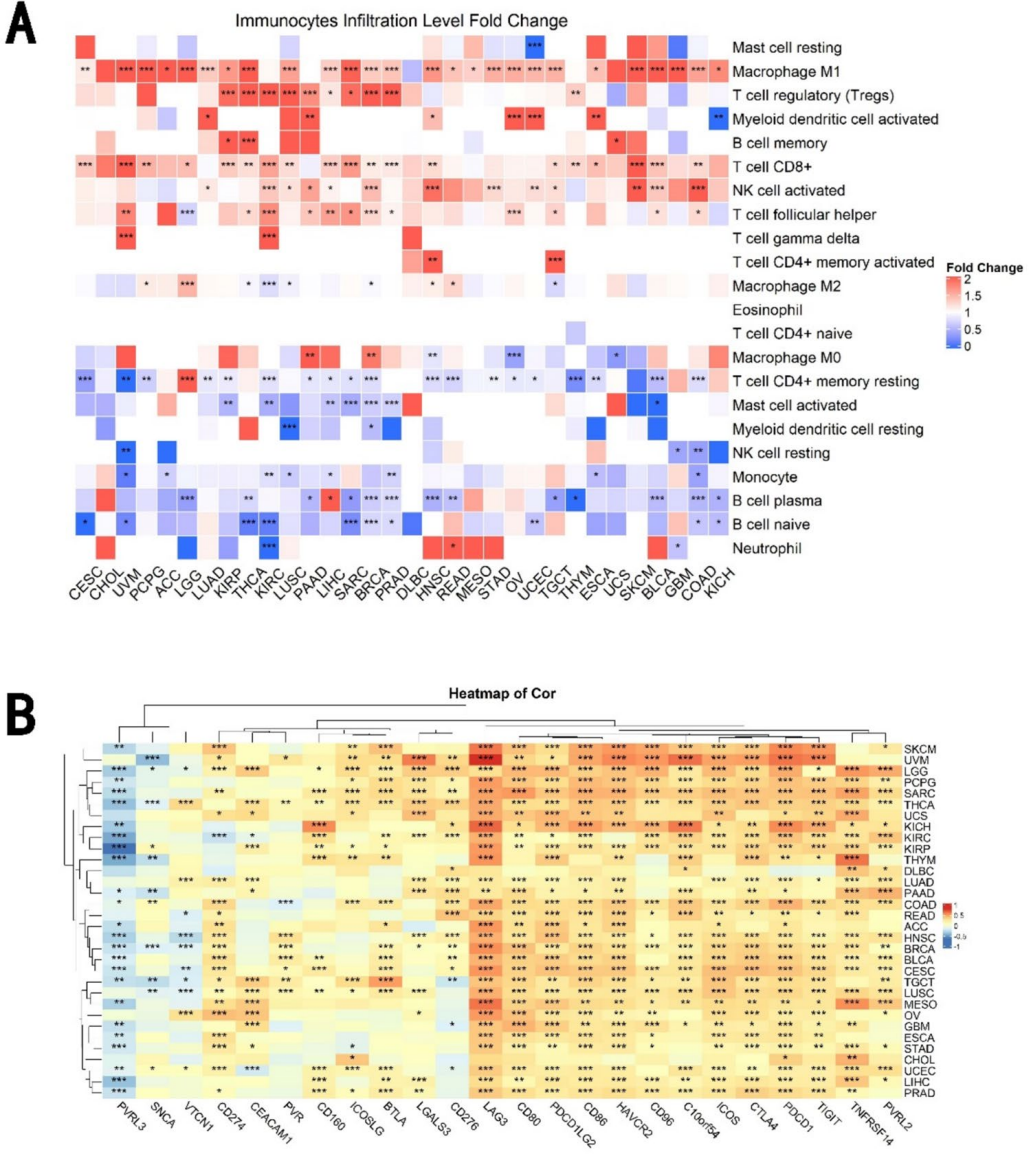
Analysis of the Correlation between ISG15 Expression Levels and Tumor Immunity. **A** Heatmap illustrating the false discovery rate (FDR)-adjusted Spearman correlation analysis between immune scores across multiple tumor tissues and ISG15 gene expression, wherein the horizontal axis represents distinct tumor tissues, the vertical axis denotes different immune scores, and various colors signify correlation coefficients. Negative values indicate an inverse correlation, while positive values denote a direct correlation. The intensity of the correlation is mirrored by the depth of the color. **B** The correlation of ISG15 expression levels with 24 immune checkpoint therapeutic markers in TCGA tumors. The p-values were adjusted using the FDR correction to account for multiple comparisons. *FDR-adjusted *p* < 0.05, **FDR-adjusted *p* < 0.01, ***FDR-adjusted *p* < 0.001; asterisks denote significance levels

We explored the relationship between ISG15, infiltrating immune cells, and stromal cells in the tumor microenvironment. ISG15 expression was positively correlated with cancer-associated fibroblast (CAF) infiltration in COAD (colon adenocarcinoma), LGG, and THCA, while showing negative correlations in several other cancers such as BRCA, CESC, HNSC (HPV-), and mesothelioma (MESO) (see Supplementary Fig. S3). We further evaluated the correlation between ISG15 expression and tumor immune cell infiltration across pan-cancer (Fig. 5A), and noticeable positive correlations were observed in STAD, BLCA, LUAD, and LUSC.

In recent years, advances have been made in immune checkpoint and cancer immunotherapy research [17]. Although PD-1 and CTLA-4 are leading targets, other immune checkpoints continue to be rigorously explored [22]. Therefore, we investigated the co-expression relationship with 24 immune checkpoints in pan-cancer, revealing that ISG15 expression generally exhibits positive correlations with a host of checkpoints and negative correlations with PVRL3 (Fig. 5B).

**Fig. 6:**
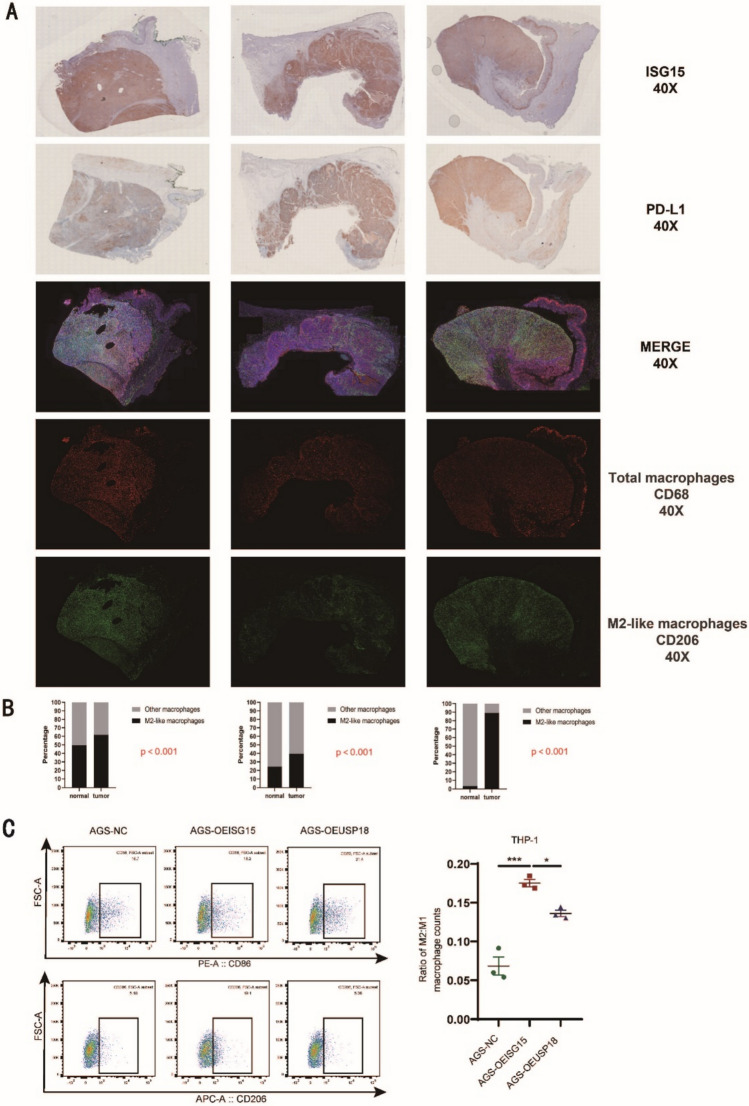
Augmented ISG15 Expression Positively Correlates With PD-L1 Expression and M2-TAM Infiltration In Gastric Cancer Tissues. **A** Multiplexed immunofluorescence and immunohistochemistry experiments were employed to detect the expression levels of ISG15 and PD-L1, as well as the infiltration levels of M2-TAMs (CD68 + /CD206 + , red + green) in cancer tissues; **B** Quantitative analysis of the comparative results of the infiltration levels of M2-TAMs in gastric cancer tissues with high ISG15 expression to those in adjacent non-cancerous tissues. **C** Flow cytometry analysis of THP-1-derived macrophages co-cultured with conditioned media AGS cells overexpressing ISG15 (AGS-OEISG15), USP18 (AGS-OEUSP18), or control cells (AGS-NC). Representative flow cytometry plots show CD86⁺ (M1) and CD206⁺ (M2) macrophage populations, with quantification of the M2:M1 macrophage ratio (right panel). Data are presented as mean ± SEM, *P < 0.05; **P < 0.01; ***P < 0.001

Moreover, employing multiplexed IF staining and IHC, we assessed ISG15 and PD-L1 expression levels, as well as M2-TAM infiltration levels, in cancer tissues from 10 gastric cancer patients from our Nanjing Drum Tower Hospital, and found a significant positive correlation among the three cases (Fig. 6A, B). To functionally validate this correlation, we performed THP-1 macrophage polarization assays using conditioned media from AGS cells overexpressing ISG15 or USP18. Flow cytometry analysis revealed that ISG15 overexpression significantly increased M2 macrophage polarization (CD206⁺) compared to the control (*p* < 0.001), while USP18 overexpression partially reversed this effect (*p* < 0.05) (Fig. 6C). Collectively, we conclude that the expression of *ISG15* and immune checkpoint genes are generally positively correlated across pan-cancers.

### ISG15 is a potential biomarker for predicting immune therapy response

**Fig. 7:**
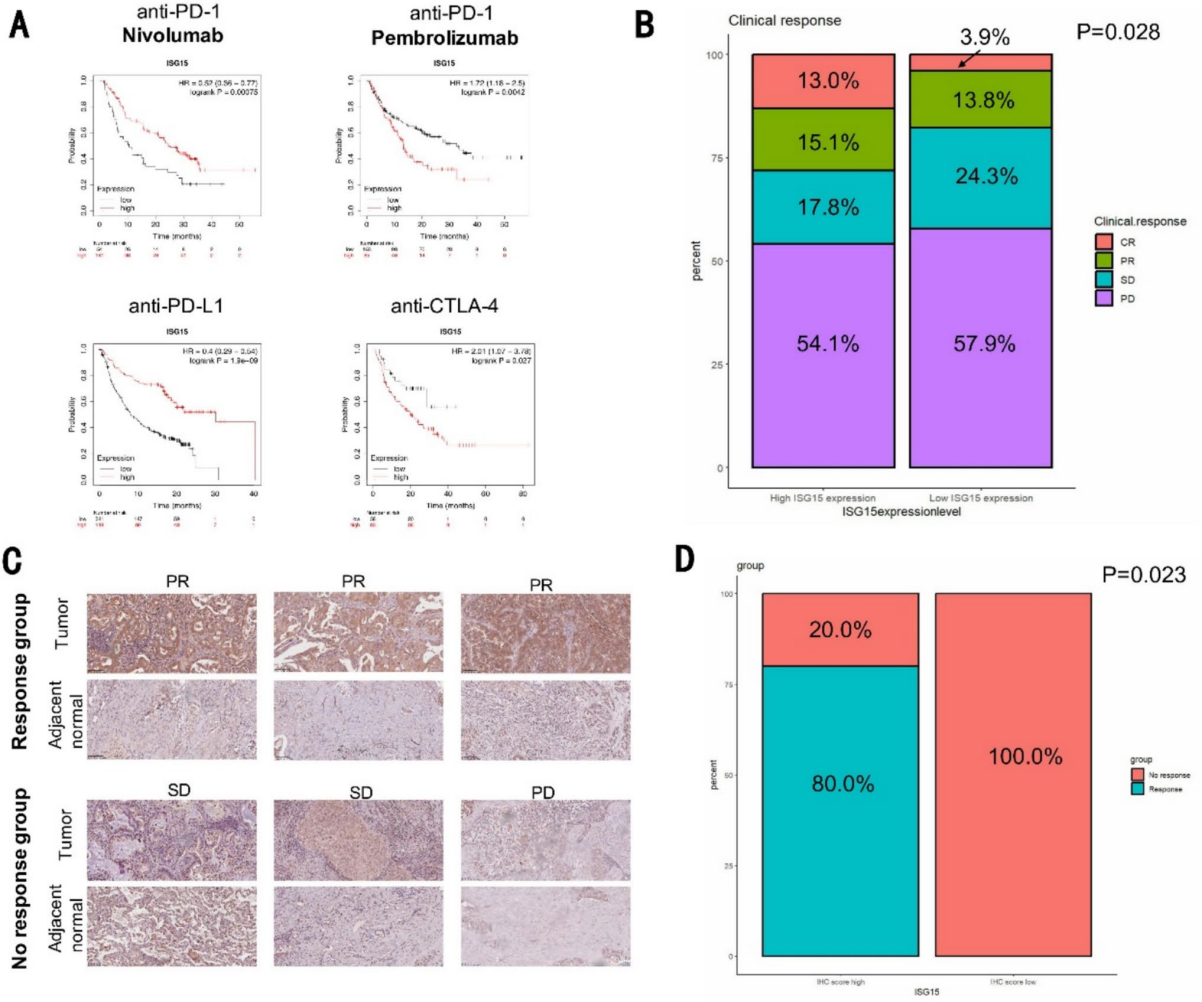
Predictive Value Of ISG15 Expression In Immunotherapy Efficacy. **A** Kaplan–Meier plotter was used to predict the response of ICB therapy. **B** Comparison of the response to anti-PD-L1 treatment in metastatic urothelial cancer patients with high and low ISG15 expression, data from the IMvigor210 dataset. **C** Protein expression (IHC) of ISG15 in anti-PD-1 response (+) patients and anti-PD-1 response (−) patients. **D** Quantitative analysis of the difference between high and low IHC scores on patient response to ICB therapy

The correlation between expression of ISG15 and immune checkpoint genes led us to investigate whether ISG15 is a biomarker for predicting the response to different types of immunotherapies. We used the Kaplan–Meier plotter (https://kmplot.com/analysis/), which provided therapy information for anti-PD-1, anti-PD-L1, and anti-CTLA-4 across nine types of cancers. Our results indicate that the patients with high expression of ISG15 had a more favorable response to anti-PD-L1 therapy (HR = 0.4, log-rank *P* = 1.9e−09) and a poorer response to anti-CTLA-4 treatment (HR = 2.01, log-rank *P* = 0.027) (Fig. 7A). In the prediction of anti-PD-1 therapeutic outcomes, high ISG15 expression indicated positive results for nivolumab but negative outcomes for pembrolizumab (Fig. 7A). Subsequently, we pursued an investigation into a larger, internationally recognized public database pertaining to PD-L1 monotherapy, which specifically focused on the IMvigor210 CoreBiologies cohort, which presents a substantial Phase 2 cohort study on the combination treatment of atezolizumab monotherapy and PD-L1 inhibitor for metastatic urothelial carcinoma (mUC). We found that patients exhibiting high ISG15 expression appear sensitive to PD-L1 inhibitor immunotherapy (Fig. 7B). To substantiate these conclusions, we selected 12 lung cancer patients undergoing anti-PD-1 therapy from the Nanjing Drum Tower Hospital for a follow-up study, and confirmed that patients with high ISG15 expression were more responsive to anti-PD-1 therapy (Fig. 7C, D).

## Discussion

ISG15 plays an oncogenic role in multiple cancers by regulating diverse mechanisms. For example, in basal breast cancer cells, ISG15 promotes tumor cell growth by maintaining the activation of the PI3K/AKT pathway through the inhibition of the formation of a complex between GDP dissociation inhibitor 2 and the epidermal growth factor receptor (EGFR) endocytic regulator Rab5 with ISGylation, thus reducing the ratio of EGFR transported to the Golgi body [7]. ISG15 is also involved in cancer cell migration by binding to and activating the Rho GTPase family member Rac1 [26], and regulating traditional EMT (epithelial–mesenchymal transition) signaling among others [27]. Current research on ISG15 in tumors is limited. Our pan-cancer pathway analyses reveal that ISG15 significantly and positively correlated to differentiation, inflammation, proliferation, metastasis, and angiogenesis, and negatively correlated with DNA repair, which may provide a general direction for future research.

ISG15 has been reported to be aberrantly overexpressed in various malignant tumors, including high-grade serous ovarian cancer [28], cervical cancer [29], breast cancer [30], and prostate cancer [31], and is associated with poor prognosis [30]. It also serves as a potential diagnostic biomarker in the serum of cancer patients [31]. Our findings are consistent with these reports with extension to additional cancer types. Concurrently, we propose that such poor prognosis might be attributed to the impact of ISG15 on immune infiltration.

Our findings have consistently shown that, in general, high expression of ISG15 has a poor prognosis. However, there were previous studies that have shown that high expression of ISG15 is associated with better prognosis in tumor patients [28, 32], and patients with ISG15 nuclear-positive features have the best prognosis and are less likely to relapse [32].These data are contradictory to our findings reported here in this work, which could be associated with CAF infiltration levels, as our immune-related screening identified a positive correlation between ISG15 expression and CAF infiltration levels in LUAD. Overall, ISG15 has been proved to regulate cancer progression and metastasis in various ways; thus, the regulatory role and prognostic predictive value of ISG15 in pan-cancers are apparent.

Since its discovery, ISG15 has been acknowledged to participate in innate immune responses, such as activating natural killer (NK) cells, enhancing the cytotoxic activity of activated lymphocytes, inducing dendritic cell maturation, and recruiting neutrophils [33]. To explore the immunomodulatory role of ISG15 in the tumor microenvironment, we conducted a comprehensive analysis of the ISG15 gene in 33 different tumors based on data from TCGA, GEO, and cohort data from Zhongshan Hospital affiliated with Fudan University and Nanjing Drum Tower Hospital affiliated with Nanjing Medical University, with regard to patient prognosis impact and immunorelevance.

In our previous research monitoring CC036 mouse stomach cancer development and its immune microenvironment, we proposed that ISG15 might mediate the immune escape microenvironment in immune inflamed stomach cancer that may promote tumor development [10]. In this study, using multicolor immunofluorescence, we detected the expression levels of ISG15 and a key component of the stomach cancer immune microenvironment, PD-L1, as well as infiltration levels of M2-TAM in human stomach cancer tissues. We found that all three elements were significantly positively correlated, i.e., stomach cancer samples with high ISG15 expression were accompanied by high PD-L1 expression and significant M2-TAM infiltration. To further investigate the functional role of ISG15 in macrophage polarization, we performed THP-1 macrophage co-culture assays. Our results demonstrated that ISG15 overexpression significantly enhanced M2 macrophage polarization (CD206⁺), whereas USP18 overexpression partially reversed this effect, indicating that ISGylation is required for this process. These findings provide functional evidence that ISG15 contributes to an immunosuppressive tumor microenvironment by promoting M2 macrophage polarization, which aligns with our clinical observations of increased M2-TAM infiltration in ISG15-high gastric cancer tissues. Meanwhile, results from both public databases and our cohort samples indicate that the expression level of ISG15 could significantly predict the therapeutic effects of anti-PD-1, anti-PD-L1, and anti-CTLA-4, primarily indicating a favorable prognosis following effective treatment. To enhance the clinical utility of ISG15, it could be integrated into multiparametric prediction models alongside established biomarkers such as PD-L1 and TMB. By combining ISG15 expression levels with other immune and clinical factors, such as immune cell infiltration and histological features, more accurate and individualized predictions of therapy response may be achieved. Furthermore, developing nomogram models incorporating ISG15 and these factors could significantly improve patient stratification for immunotherapy and guide personalized treatment strategies. We believe that this provides a more sensitive and specific immunotherapeutic efficacy prediction biomarker, which is of immense significance clinically. To further validate the prognostic and predictive roles of ISG15, future studies should focus on expanding the cohort size and including prospective clinical trials to assess its utility as a biomarker in various cancers. Experimental studies using animal models and patient-derived organoids will be essential to elucidate the underlying mechanisms through which ISG15 regulates immune infiltration and impacts therapy outcomes. Moreover, the development of standardized assays for detecting ISG15 in clinical samples will be a critical step toward its application in routine diagnostics. It should be pointed out that, other immune checkpoints in different tumors, and their relationship with ISG15, also warrant further in-depth research.

Here, we have described the prognostic value of ISG15 in pan-cancer and reported its immunological relevance in various cancers. As aforementioned, ISG15 interacts intracellularly with target proteins through covalent or non-covalent modifications, or participates in a number of biological processes, playing a vital role in the tumor immune microenvironment. However, a clear conclusion on the relationship between ISG15 and tumor immune infiltration has yet to be seen. We were surprised to find a significant correlation between the two in STAD (stomach adenocarcinoma), LUAD (lung adenocarcinoma), CHOL (cholangiocarcinoma), BRCA (breast invasive carcinoma), and BLCA (bladder urothelial carcinoma). We validated these results in certain tumors using multicenter clinical samples. These studies may provide new insights for subsequent research for further establishing the relationship between ISG15 and immune microenvironment. While our findings highlight the potential of ISG15 as a biomarker for immunotherapy, its clinical application also raises ethical and social considerations. From an ethical perspective, ensuring patient consent and protecting data privacy will be paramount as ISG15-based diagnostics become integrated into clinical practice. Socially, it is crucial to address the accessibility and affordability of ISG15 testing to ensure equitable implementation, particularly in resource-limited settings. These considerations will be critical in translating ISG15 into a clinically applicable biomarker while minimizing disparities in healthcare access.

## Conclusion

Our findings have shown that the expression level of ISG15 in most tumors is higher than in normal tissues. An increase in ISG15 expression typically indicates a poor prognosis for patients. We also observed varying degrees of correlation between ISG15 and immune infiltration across different tumors. In addition, we demonstrated that ISG15 is associated with tumor immune infiltration and may impact immunotherapy across pan-cancer. To the best of our knowledge, this is the first report on the correlation between ISG15, pan-cancer immune infiltration, and responses to different immunotherapies.

## Supplementary Information

Below is the link to the electronic supplementary material.

Supplementary file1 (DOCX 2283 KB)

## Data Availability

The datasets analyzed in this study are available from publicly accessible sources. The transcriptomic and genomic data were obtained from The Cancer Genome Atlas (TCGA) (https://portal.gdc.cancer.gov/) and Gene Expression Omnibus (GEO) (https://www.ncbi.nlm.nih.gov/geo/). The IMvigor210 cohort data used for immunotherapy response analysis were obtained from (http://research-pub.gene.com/IMvigor210CoreBiologies). Clinical validation data from patient cohorts at Nanjing Drum Tower Hospital are available upon reasonable request from the corresponding author. Additional data supporting the findings of this study are included in the supplementary information.

## Abbreviations

AML: Acute myeloid leukemia
BLCA: Bladder urothelial carcinoma
BRCA: Breast invasive carcinoma
CAF: Cancer-associated fibroblast
CAR-T: Chimeric antigen receptor t cells
CC: Collaborative cross
CESC: Cervical squamous cell carcinoma and endocervical adenocarcinoma
CHOL: Cholangiocarcinoma
COAD: Colon adenocarcinoma
CR: Complete remission
CTLA-4: Cytotoxic t-lymphocyte antigen 4
DFS: Disease-free survival
DMFS: Distant metastasis-free survival
DSS: Disease-specific survival
EFS: Event-free survival
EGFR: Epidermal growth factor receptor
EMT: Epithelial-mesenchymal transition
ESCA: Esophageal carcinoma
GBM: Glioblastoma multiforme
HNSC: Head and neck squamous cell carcinoma
ICB: Immune checkpoint blockade
ICI: Immune checkpoint inhibitors
IF: Immunofluorescence
IFN: Type I interferon
IHC: Immunohistochemistry
ISG15: Interferon-stimulated gene 15
KICH: Kidney chromophobe
KIRC: Kidney renal clear cell carcinoma
KIRP: Kidney renal papillary cell carcinoma
LGG: Brain lower grade glioma
LIHC: Liver hepatocellular carcinoma
LUAD: Lung adenocarcinoma
LUSC: Lung squamous cell carcinoma
M2-TAM: M2-like tumor-associated macrophage
MESO: Mesothelioma
MMR: Mis-match repair
MSI-H: Microsatellite instability-high
mUC: Metastatic urothelial carcinoma
NK: Natural killer
OS: Overall survival
PAAD: Pancreatic adenocarcinoma
PCPG: Pheochromocytoma and paraganglioma
PD: Progressive disease
PD1: Programmed cell death protein 1
PD-L1: Programmed cell death ligand 1
PFS: Progression-free survival
PPS: Post-progression survival
PR: Partial response
PRAD: Prostate adenocarcinoma
RB: Retinoblastoma
RFS: Relapse-free survival
SARC: Sarcoma
SD: Stable disease
SKCM: Skin cutaneous melanoma
STAD: Stomach adenocarcinoma
TAMs: Tumor-associated macrophages
THCA: Thyroid carcinoma
TMB: Tumor mutation burden
TME: Tumor microenvironment
Tregs: Regulatory t cells
t-SNE: T-distributed stochastic neighbor embedding
UB31L: Ubiquitin-activating enzyme E1
UCEC: Uterine corpus endometrial carcinoma
UM: Uveal melanoma
USP18: Ubiquitin-specific peptidase
UVM: Uveal melanoma

## References

[1] Blomstrom DC, Fahey D, Kutny R, Korant BD, Knight E (1986) Molecular characterization of the interferon-induced 15-kDa protein. Molecular cloning and nucleotide and amino acid sequence. J Biol Chem 261(19):8811–8816 3087979

[2] Jinawath N et al (2004) Comparison of gene-expression profiles between diffuse- and intestinal-type gastric cancers using a genome-wide cDNA microarray. Oncogene 23(40):6830–6844 15273739 10.1038/sj.onc.1207886

[3] Mirzalieva O, Juncker M, Schwartzenburg J, Desai S (2022) ISG15 and ISGylation in human diseases. Cells 11(3):538 35159348 10.3390/cells11030538 PMC8834048

[4] Moro RN et al (2023) Interferon restores replication fork stability and cell viability in BRCA-defective cells via ISG15. Nat Commun 14(1):6140 37783689 10.1038/s41467-023-41801-w PMC10545780

[5] Thery F, Eggermont D, Impens F (2021) Proteomics mapping of the ISGylation landscape in innate immunity. Front Immunol 12:720–765 10.3389/fimmu.2021.720765 PMC8383068 34447387

[6] Han HG, Moon HW, Jeon YJ (2018) ISG15 in cancer: beyond ubiquitin-like protein. Cancer Lett 438:52–62 30213559 10.1016/j.canlet.2018.09.007

[7] Bolado-Carrancio A et al (2021) ISGylation drives basal breast tumour progression by promoting EGFR recycling and Akt signalling. Oncogene 40(44):6235–6247 34556814 10.1038/s41388-021-02017-8 PMC8566238

[8] Grivennikov SI, Greten FR, Karin M (2010) Immunity, inflammation, and cancer. Cell 140(6):883–899 20303878 10.1016/j.cell.2010.01.025 PMC2866629

[9] Noll KE, Ferris MT, Heise MT (2019) The collaborative cross: a systems genetics resource for studying host-pathogen interactions. Cell Host Microbe 25(4):484–498 30974083 10.1016/j.chom.2019.03.009 PMC6494101

[10] Wang P et al (2019) Diverse tumour susceptibility in Collaborative Cross mice: identification of a new mouse model for human gastric tumourigenesis. Gut 68(11):1942–1952 30842212 10.1136/gutjnl-2018-316691 PMC6839736

[11] Nguyen H-M et al (2023) Interferon stimulated gene 15 (ISG15) in cancer: an update. Cancer Lett 556:216080 36736853 10.1016/j.canlet.2023.216080

[12] Huang H-W et al (2021) Association between inflammation and function of cell adhesion molecules influence on gastrointestinal cancer development. Cells 10(1):67 33406733 10.3390/cells10010067 PMC7824562

[13] Li X et al (2023) The immune escape mechanism of nasopharyngeal carcinoma. FASEB J 37(7):e23055 37358482 10.1096/fj.202201628RR

[14] Chen DS, Mellman I (2017) Elements of cancer immunity and the cancer-immune set point. Nature 541(7637):321–330 28102259 10.1038/nature21349

[15] Li B, Chan HL, Chen P (2019) Immune checkpoint inhibitors: basics and challenges. Curr Med Chem 26(17):3009–3025 28782469 10.2174/0929867324666170804143706

[16] Fridman WH, Pagès F, Sautès-Fridman C, Galon J (2012) The immune contexture in human tumours: impact on clinical outcome. Nat Rev Cancer 12(4):298–306 22419253 10.1038/nrc3245

[17] Lazăr DC et al (2018) Prognostic significance of tumor immune microenvironment and immunotherapy: novel insights and future perspectives in gastric cancer. World J Gastroenterol 24(32):3583–3616 30166856 10.3748/wjg.v24.i32.3583 PMC6113718

[18] Zhang C et al (2021) Immune landscape of gastric carcinoma tumor microenvironment identifies a peritoneal relapse relevant immune signature. Front Immunol 12:6510–6533 10.3389/fimmu.2021.651033 PMC8155484 34054812

[19] Fuchs CS et al (2022) Pembrolizumab versus paclitaxel for previously treated PD-L1-positive advanced gastric or gastroesophageal junction cancer: 2-year update of the randomized phase 3 KEYNOTE-061 trial. Gastr Cancer 25(1):197–206 10.1007/s10120-021-01227-z PMC8732941 34468869

[20] Balar AV et al (2017) Atezolizumab as first-line treatment in cisplatin-ineligible patients with locally advanced and metastatic urothelial carcinoma: a single-arm, multicentre, phase 2 trial. Lancet 389(10064):67–76 27939400 10.1016/S0140-6736(16)32455-2 PMC5568632

[21] Harada K, Baba H, Ajani JA (2018) Recent trend in gastric cancer treatment in the USA. J Cancer Metastasis Treat 4:18 34113719 10.20517/2394-4722.2017.74 PMC8188734

[22] Joshi SS, Badgwell BD (2021) Current treatment and recent progress in gastric cancer. CA Cancer J Clin 71(3):264–279 33592120 10.3322/caac.21657 PMC9927927

[23] Chao J, Fuchs CS, Shitara K, Tabernero J (2020) Pembrolizumab (pembro) in microsatellite instability-high (MSI-H) advanced gastric/gastroesophageal junction (G/GEJ) cancer by line of therapy. J Clin Oncol 38(4):430

[24] Yuan B et al (2022) A novel DNA repair gene signature for immune checkpoint inhibitor-based therapy in gastric cancer. Front Cell Dev Biol 10:893546 35676932 10.3389/fcell.2022.893546 PMC9168368

[25] Gibney GT, Weiner LM, Atkins MB (2016) Predictive biomarkers for checkpoint inhibitor-based immunotherapy. Lancet Oncol 17(12):e542–e551 27924752 10.1016/S1470-2045(16)30406-5 PMC5702534

[26] Chen Y-L et al (2019) Interferon-stimulated gene 15 modulates cell migration by interacting with Rac1 and contributes to lymph node metastasis of oral squamous cell carcinoma cells. Oncogene 38(23):4480–4495 30765861 10.1038/s41388-019-0731-8

[27] Song F et al (2021) Identification of novel key genes associated with the metastasis of prostate cancer based on bioinformatics prediction and validation. Cancer Cell Int 21(1):559 34696780 10.1186/s12935-021-02258-3 PMC8547030

[28] Yeung T-L et al (2018) ISG15 promotes ERK1 ISGylation, CD8+ T cell activation and suppresses ovarian cancer progression. Cancers (Basel) 10(12):464 30469497 10.3390/cancers10120464 PMC6316352

[29] Tao P, Sun L, Sun Y, Wang Y (2022) ISG15 is associated with cervical cancer development. Oncol Lett 24(4):380 36238852 10.3892/ol.2022.13500 PMC9494601

[30] Kariri YA et al (2021) The prognostic significance of interferon-stimulated gene 15 (ISG15) in invasive breast cancer. Breast Cancer Res Treat 185(2):293–305 33073304 10.1007/s10549-020-05955-1 PMC7867506

[31] Zuo D et al (2021) Identification of hub genes and their novel diagnostic and prognostic significance in pancreatic adenocarcinoma. Cancer Biol Med 19(7):1029–1046 34403221 10.20892/j.issn.2095-3941.2020.0516 PMC9334760

[32] Qu T et al (2020) ISG15 induces ESRP1 to inhibit lung adenocarcinoma progression. Cell Death Dis 11(7):511 32641707 10.1038/s41419-020-2706-7 PMC7343783

[33] Kang JA, Kim YJ, Jeon YJ (2022) The diverse repertoire of ISG15: more intricate than initially thought. Exp Mol Med 54(11):1779–1792 36319753 10.1038/s12276-022-00872-3 PMC9722776

