# Supplementary material for: Cohort-based pan-cancer analysis and experimental studies reveal ISG15 gene as a novel biomarker for prognosis and immunotherapy efficacy prediction
Source: Cancer Immunol Immunother. 2025 Apr 10;74(5):168. doi: 10.1007/s00262-025-04026-y (PMC11985735; doi:10.1007/s00262-025-04026-y)

**Supplementary Methods**

1. **Gene and Protein Expression Analysis**
   To analyze the expression levels of ISG15 in various tumor and normal tissues, we procured TCGA-PanCancer data from UCSC Xena (<https://xena.ucsc.edu/>) and performed rank-sum tests to evaluate mRNA expression differences. Using the "Gene_DE" module on the TIMER2 website (<http://timer.cistrome.org/>), we compared ISG15 expression levels in tumor and adjacent normal tissues. For tumors lacking paired normal tissue data in TIMER2, we supplemented the analysis with GTEx data from the GEPIA2 website (<http://gepia2.cancer-pku.cn/#index>). At the protein level, differential expression of ISG15 was assessed using the "CPTAC" module on UALCAN (<https://ualcan.path.uab.edu/>). Additionally, immunohistochemical images of tumors and corresponding normal tissues were obtained from the Human Protein Atlas (HPA, <https://www.proteinatlas.org/>) to further validate the protein expression differences.
2. **Gene Mutation and Copy Number Variation Analysis**
   All pan-cancer gene mutation information was obtained from cBioPortal (<http://www.cbioportal.org/>). We analyzed the proportions of various mutation types present in ISG15 across cancers. Kaplan-Meier survival curves for overall survival, progression-free survival, and disease-free survival were generated to assess the prognostic impact of ISG15 mutations. Mutation data from gist2 within the TCGA-PanCancer dataset, obtained from UCSC Xena, were used to analyze ISG15 copy number variations (CNVs) in different cancers. Statistical analyses included the Mann-Whitney U test to compare CNV frequencies and Spearman correlation to evaluate relationships between ISG15 mRNA expression levels and CNVs. Median expression levels of ISG15 across three gene variation groups (gain, loss, no change) were compared using rank-sum tests.
3. **Single-cell Level Tumor Functional Status Analysis**
   Single-cell sequencing data were obtained from the CancerSEA website (<http://biocc.hrbmu.edu.cn/CancerSEA/home.jsp>), which includes 72 single-cell datasets totaling 41,900 cancer cells across 25 human cancer types. By searching for the ISG15 gene in CancerSEA, we retrieved data on ISG15 expression and its correlation with 14 tumor functional states, including proliferation, apoptosis, angiogenesis, and invasion. We specifically demonstrated six significantly correlated functional states in Acute Myeloid Leukemia (AML), Retinoblastoma (RB), and Uveal Melanoma (UM). Single-cell t-SNE plots were downloaded directly from CancerSEA to display the expression distribution of ISG15. The t-SNE visualizations were processed using CancerSEA's built-in tools without additional filtering.
4. **Survival Prognosis Analysis**
   Survival analysis was conducted using the “Survival Analysis-Survival Map” module on GEPIA2 (<http://gepia2.cancer-pku.cn/#index>) to evaluate overall survival (OS) and disease-free survival (DFS) across TCGA cancers. Tumors with significant OS or DFS differences (p < 0.05) were selected for Kaplan-Meier survival curve analysis using a 50% cutoff to distinguish between high and low ISG15 expression groups. The “Pathological Stage Plot” module of GEPIA2 was employed to visualize ISG15 expression across pathological stages using violin plots. Validation was performed using Kaplan-Meier Plotter (<https://kmplot.com/analysis/>) for meta-gene chip data from eight tumors, where the best cutoff values for prognostic indicators were applied. Survival analysis for 244 intrahepatic cholangiocarcinoma patients from Zhongshan Hospital of Fudan University ^1^ was conducted using the Kaplan-Meier method, with a follow-up period exceeding five years. Statistical significance was determined by the log-rank test, with p < 0.05 considered significant.
5. **Immune Infiltration Analysis**
   Immune infiltration analyses were conducted using the “Immune-Gene” module of TIMER2 (<http://timer.cistrome.org/>), with Spearman’s rank correlation adjusted for tumor purity to examine ISG15 expression and cancer-associated fibroblast infiltration across cancers. Results were visualized as heatmaps and scatter plots. Immune cell infiltration levels were estimated using the CIBERSORT algorithm within TCGA-PanCancer data, with fold changes calculated as median immune cell infiltration levels in the high-expression group divided by those in the low-expression group. Rank-sum tests were used to assess statistical differences in infiltration levels between the two groups. ISG15 correlations with 24 immune checkpoint therapeutic markers were analyzed using Spearman Correlation Analysis, and p-values were adjusted for multiple comparisons using the Benjamini-Hochberg FDR correction. Cluster analysis was performed to examine ISG15 expression across different immune subtypes. For immune checkpoint blockade (ICB)-related genes, data were referenced from Golnaz Morad et al.^2^
6. **Relationship Between ISG15 and Immunotherapy Outcomes**

Immunotherapy outcomes were analyzed using data from Kaplan-Meier Plotter and IMvigor210 datasets. Anti-PD-1 therapy data included 195 patients treated with nivolumab and 238 with pembrolizumab. Anti-PD-L1 data included 459 patients, and anti-CTLA-4 data included 121 patients. Survival curves were generated to assess the impact of ISG15 expression on overall survival (OS) in nine cancer types. Surgical lung cancer tissues from Nanjing Drum Tower Hospital were analyzed for ISG15 expression using immunohistochemistry (IHC). IHC scores were calculated using the formula: IHC score = Σ (proportion of stained intensity cells × staining intensity score), with staining intensity scored as 0 (negative), 1 (weakly positive), 2 (moderately positive), and 3 (strongly positive), and staining range scored as 0 (no staining), 1 (1%-25%), 2 (26%-50%), 3 (51%-75%), and 4 (76%-100%). Patients were categorized into high and low ISG15 expression groups using a cutoff of 50% IHC score. Treatment efficacy between these groups was compared using chi-square tests. For the IMvigor210 dataset, ISG15 expression was analyzed in relation to four immunotherapeutic responses (complete response, partial response, stable disease, and progressive disease), with statistical significance determined using chi-square tests.

1. **Immunohistochemistry (IHC) and Multiplexed Immunofluorescence (IF) Staining**

The specificity of the ISG15 antibody was verified using Western blot analysis with cell lysates from the human gastric cancer cell line AGS (Shanghai Institute of Biochemistry and Cell Biology, Shanghai, China) and the 293T cell line as a positive control. The rabbit anti-ISG15 antibody (1:1000) was incubated overnight at 4°C, followed by an appropriate HRP-conjugated secondary antibody (7074, Cell Signaling, USA, 1:3000). Bands were detected using the High Sensitivity ECL Kit (HY-K2005, MedChemExpress LLC, China), with a specific band observed at approximately 15 kDa, consistent with the expected molecular weight of ISG15. β-actin was used as a loading control, showing stable expression across all lanes (Supplementary Fig. 4).
With informed consent from 12 lung cancer patients and 10 gastric cancer patients, tissue samples were obtained from the Department of Pathology at the Nanjing Drum Tower Hospital. Paraffin sections were baked at 60-65°C for 3 hours, followed by thorough dewaxing and hydration utilizing xylene and ethanol with descending concentrations. Antigen retrieval was performed using a citrate buffer solution (pH 6.0) through microwave technology at 92-95°C for a total of 15 minutes, and the sections were allowed to cool to room temperature. Hydrogen peroxide blocking solution was applied for 10 minutes to inactivate endogenous peroxidase. Slides were blocked with goat serum for 15-30 minutes, followed by incubation with primary antibodies overnight at 4°C: ISG15 (Proteintech, 1:200 for lung cancer; Santa Cruz, 1:100 for gastric cancer) and PD-L1 (Proteintech, 1:100 for gastric cancer). Secondary antibodies were incubated for 2 hours at room temperature, and sections were stained with DAB, counterstained with hematoxylin, dehydrated, and coverslipped. Multiplexed IF staining followed similar steps, with modifications including fluorescently-labeled secondary antibodies, nuclear counterstaining with DAPI, and CD68 (Cell Signaling Technology, 1:400) and CD206 (Cell Signaling Technology, 1:400) markers. Stained sections were scanned using the PerkinElmer Vectra Polaris Automated Quantitative Pathology Imaging System, and data were analyzed with Inform v2.6.0 software.

1. **Data Processing and Statistical Analysis**
   Tumor data from TCGA were normalized using the FPKM (Fragments Per Kilobase Million) method. Data from the IMvigor210 dataset, comprising 348 patients with metastatic urothelial carcinoma treated with atezolizumab monotherapy, were normalized using CPM (Counts Per Million) via the edgeR package. Prior to univariate and multivariate Cox regression analyses, data cleaning was performed by removing missing values, resulting in the exclusion of 2 cases in KIRC and 62 cases in SKCM. No further screening, filtering, or batch correction was applied, and gene enrichment analysis was not performed.
   Statistical analyses included the Wilcoxon Mann-Whitney rank-sum test for group comparisons and Spearman correlation for variable associations. Group frequencies were compared using the Mann-Whitney U test and chi-square test. Kaplan-Meier survival analysis was conducted using the survival package in R, and univariate and multivariate Cox regression analyses were used to identify prognostic factors. Covariates included in the Cox regression models were as follows:

**KIRC**: age, gender, tumor stage (T), metastasis (M), pathologic stage, histologic grade, and ISG15 expression.

**SKCM**: age, gender, tumor stage (T, N, M), pathologic stage, and ISG15 expression.
A nomogram prognostic model was constructed based on the Cox regression results. All analyses were performed using R (version 4.0.2) and SPSS (version 26.0). The R packages used included survival and edgeR. Significance thresholds were set at p < 0.05 (*p < 0.05, **p < 0.01, ***p < 0.001, ****p < 0.0001).

**References**

1. Dong, L. *et al.* Proteogenomic characterization identifies clinically relevant subgroups of intrahepatic cholangiocarcinoma. *Cancer Cell* 40(1), 70–87 (2022).
2. Morad, G., Helmink, B. A., Sharma, P. & Wargo, J. A. Hallmarks of response, resistance, and toxicity to immune checkpoint blockade. *Cell* 184(21), 5309–5337 (2021).


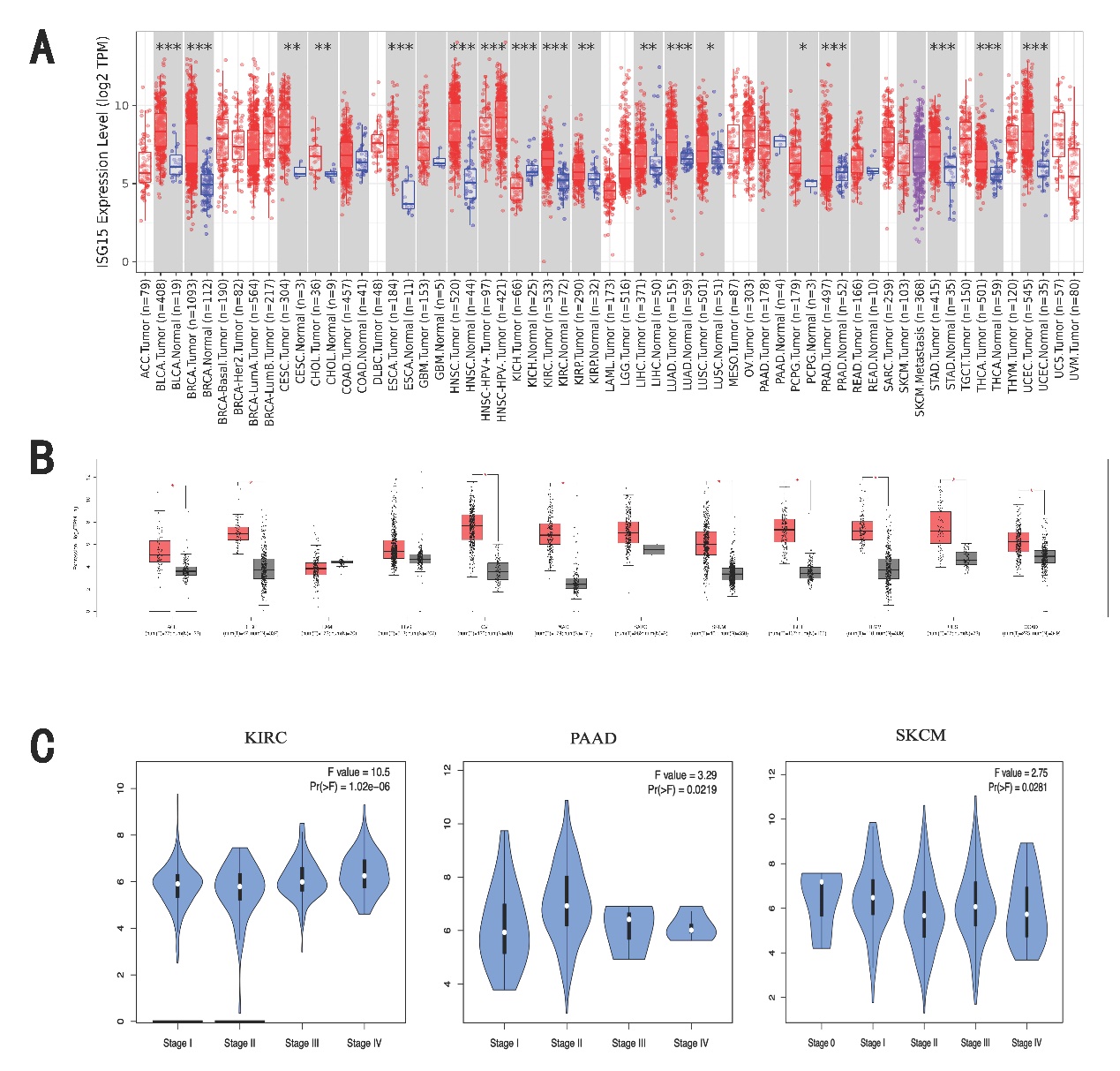


**Supplementary Figure S1. Differential Expression of ISG15 Gene Across Various Tumors and Pathological Stages.** (A) Differential mRNA expression of ISG15 between cancerous and normal tissues was interrogated utilizing TIMER2. (B) A supplementary analysis of ISG15 mRNA levels was conducted for select tumors lacking corresponding normal tissue data within TCGA; here, normal tissue data were sourced from the GTEx database. (C) ISG15 expression levels were compared across distinct pathological stages (stages I–IV) in KIRC, PAAD, and SKCM. *P < 0.05; **P < 0.01; ***P < 0.001; ****P < 0.0001.


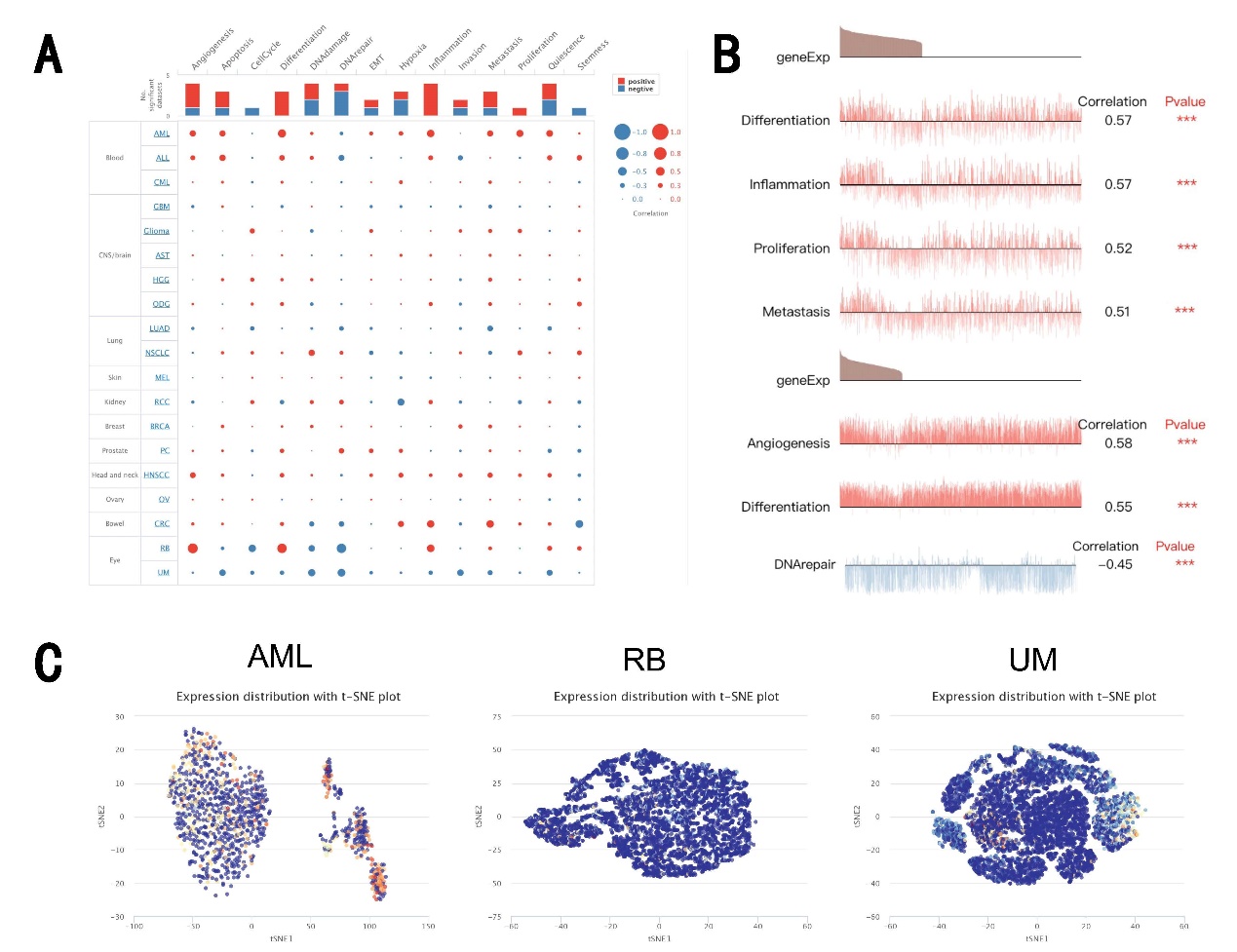


**Supplementary Figure S2. ISG15 Expression at the Single-Cell Level and Its Impact on Various Functional States of Cancer Cells.** (A) Association between ISG15 expression and diverse tumor functional states. (B) The six significantly associated functional states. (C) ISG15 expression profiles at a single-cell level in AML, RB, and UM, visualized by T-SNE maps.


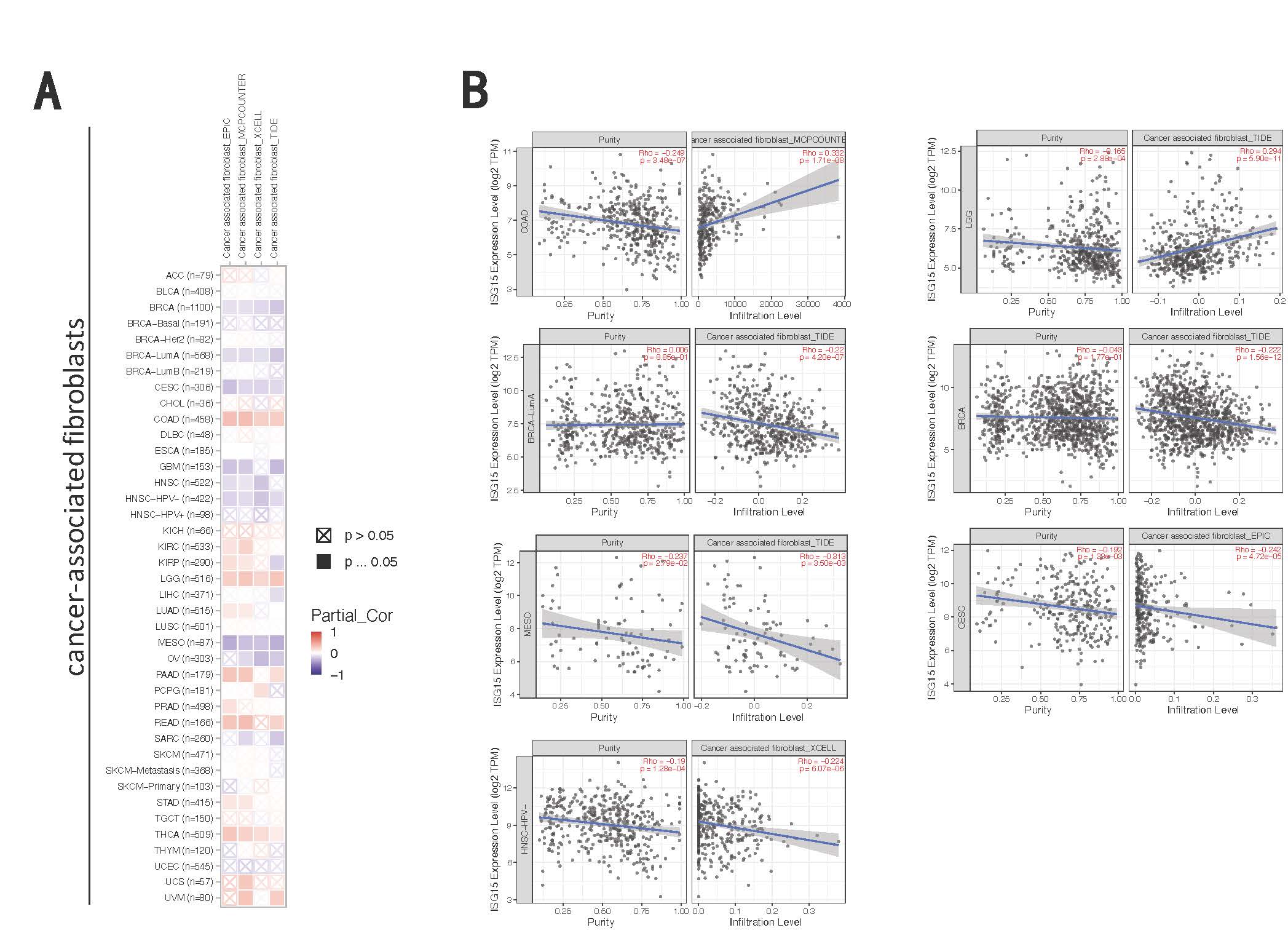


**Supplementary Figure S3. Statistical Correlations between ISG15 Expression and Cancer-Associated Fibroblast Infiltration.** (A) Associations between ISG15 expression levels across various tumors and the degree of cancer-associated fibroblast infiltration were determined utilizing algorithms EPIC, MCPCOUNTER, XCELL, and TIDE. (B) Scatter plots illustrating the correlation between ISG15 expression levels, tumor purity, and immune infiltration levels in COAD, LGG, BRCA, BRCA-LumA, MESO, CESC, and HNSC-HPV-.

**
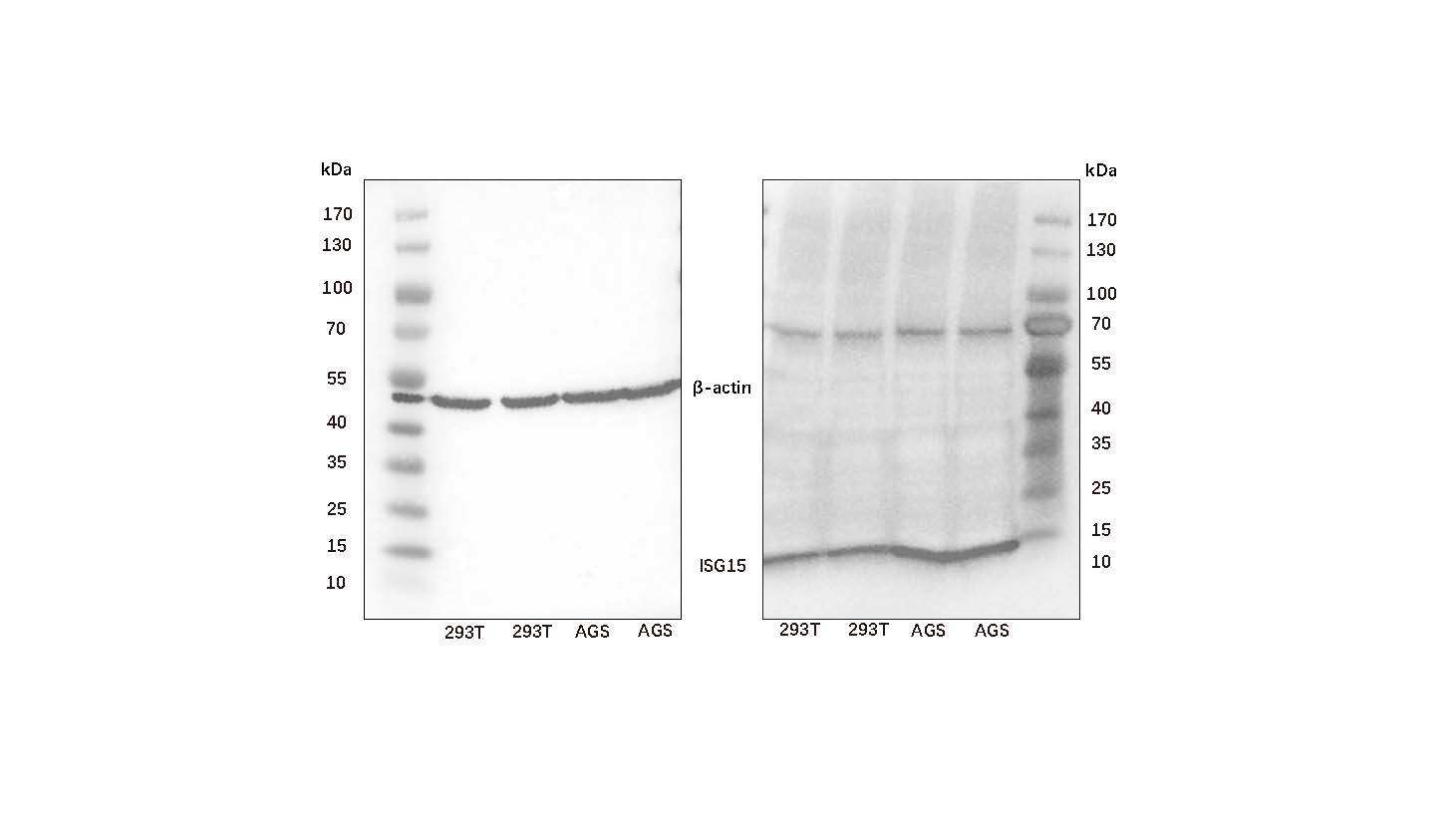
**

**Supplementary Figure S4. Validation of ISG15 Antibody Specificity by Western Blot Analysis.** Western blot analysis was performed using cell lysates from the human gastric cancer cell line AGS and the 293T cell line as a positive control. The ISG15 antibody (1:1000) detected a specific band at approximately 15 kDa, corresponding to the expected molecular weight of ISG15. β-actin was used as a loading control, confirming equal protein loading across all lanes.

**Supplementary Table S1.** ISG15 expression in the patients with different copy number changes of ISG15. The p-values were obtained by Mann-Whitney test. *P < 0.05; **P < 0.01; ***P < 0.001


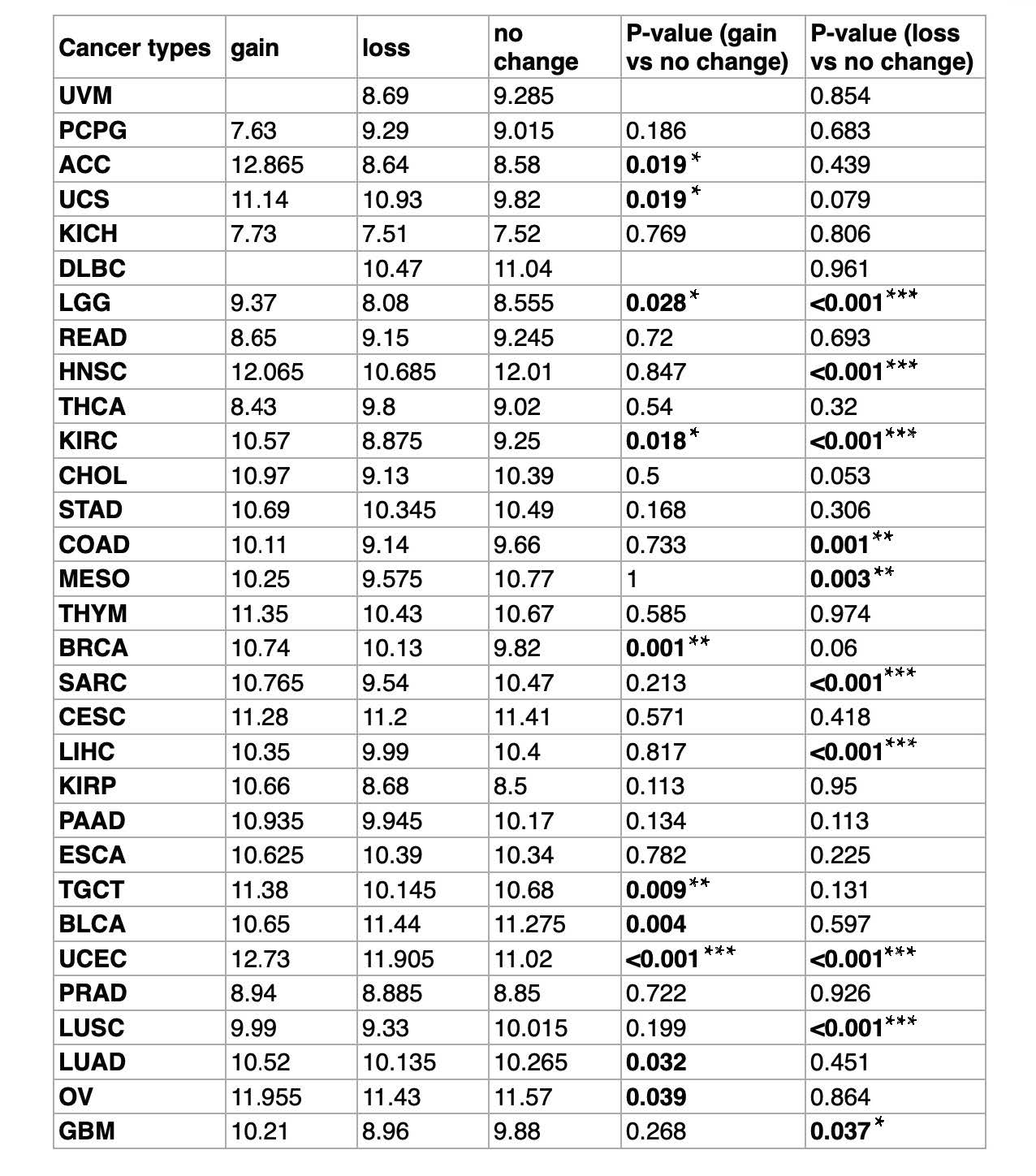


**Supplementary Table S2.** Univariate and multivariate Cox regression analysis for OS in KIRC.


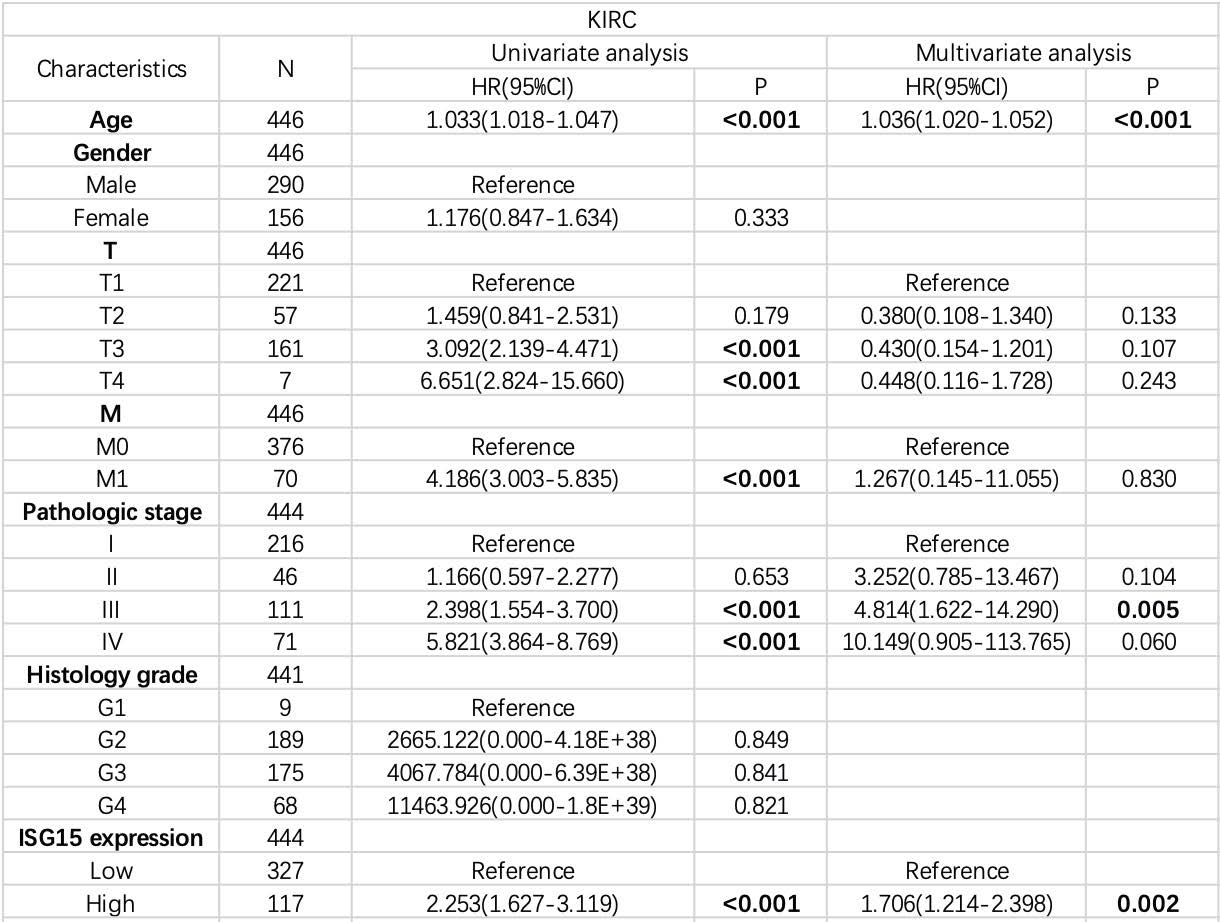


**Supplementary Table S3.** Univariate and multivariate Cox regression analysis for OS in SKCM.


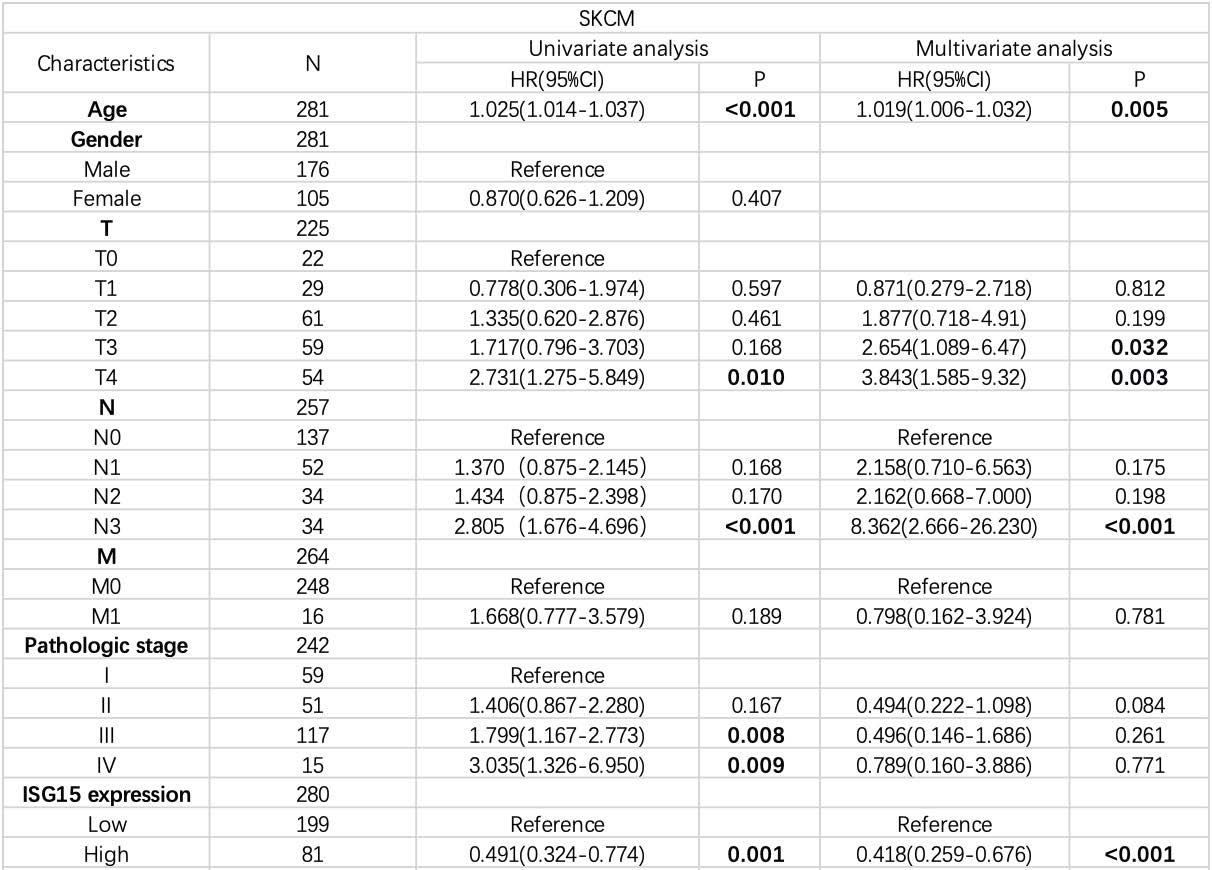


**Supplementary Table S4.** Univariate and multivariate Cox regression analysis for OS in BRCA.


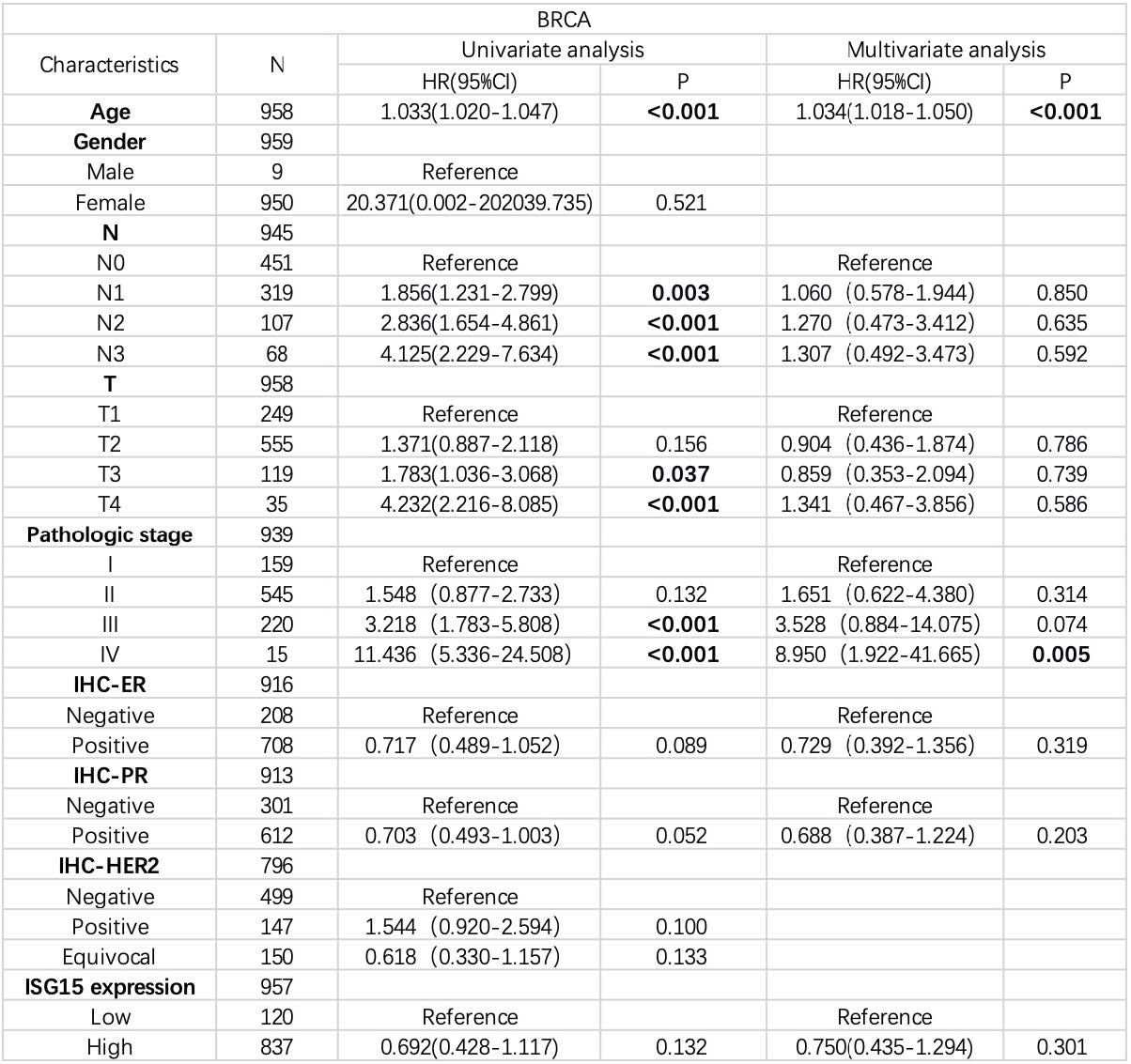


**Supplementary Table S5.** Univariate and multivariate Cox regression analysis for OS in LAML.


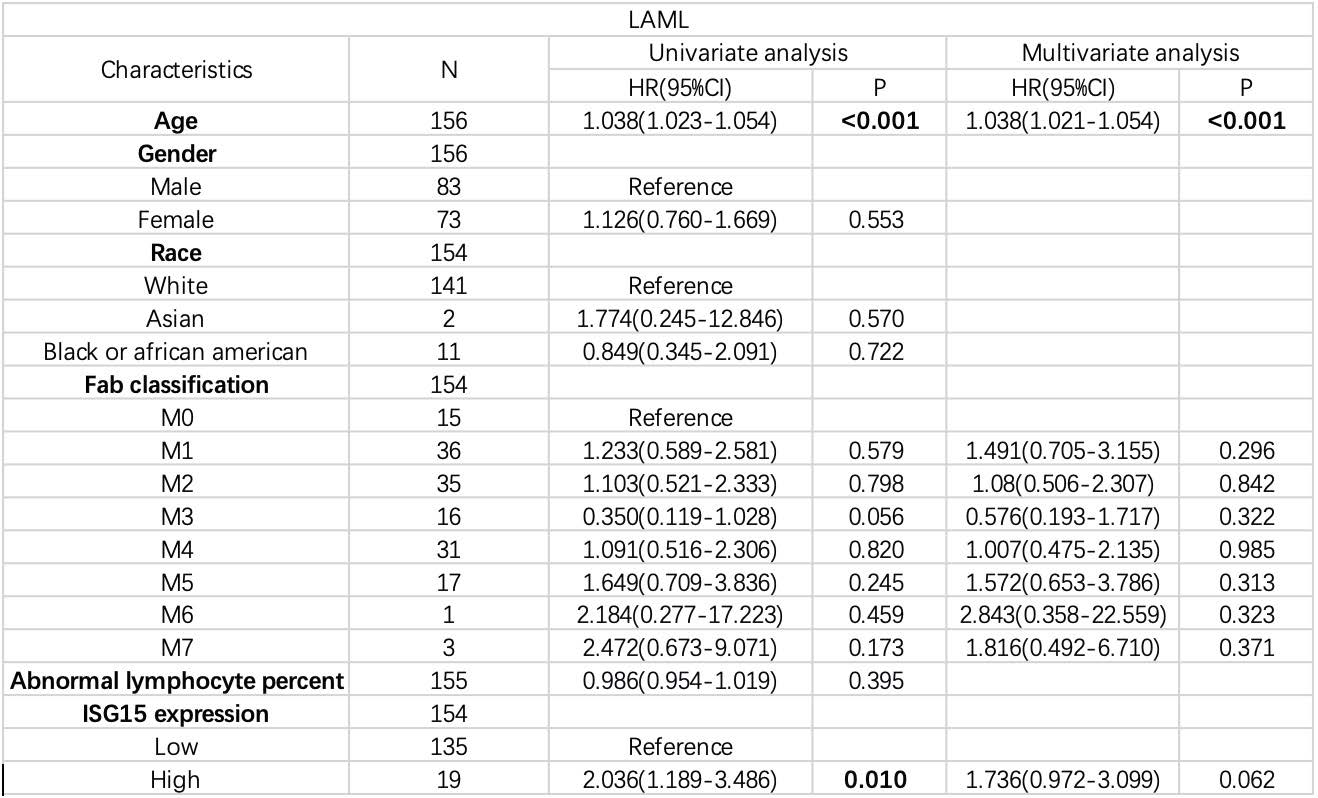


**Supplementary Table S6.** Univariate and multivariate Cox regression analysis for OS in PAAD.


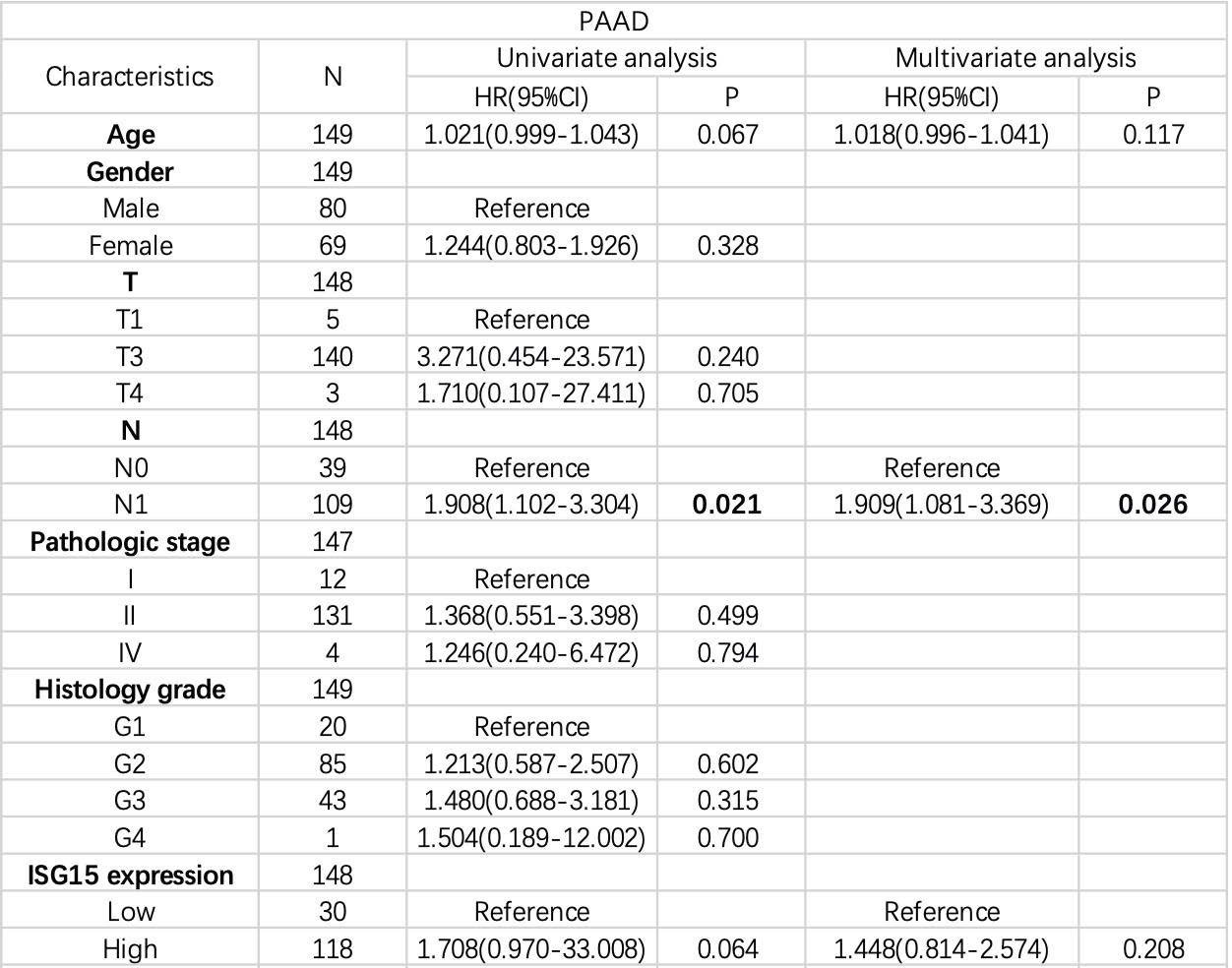


**Supplementary Table S7.** Association of ISG15 with 14 Functional States Across Various Cancers.


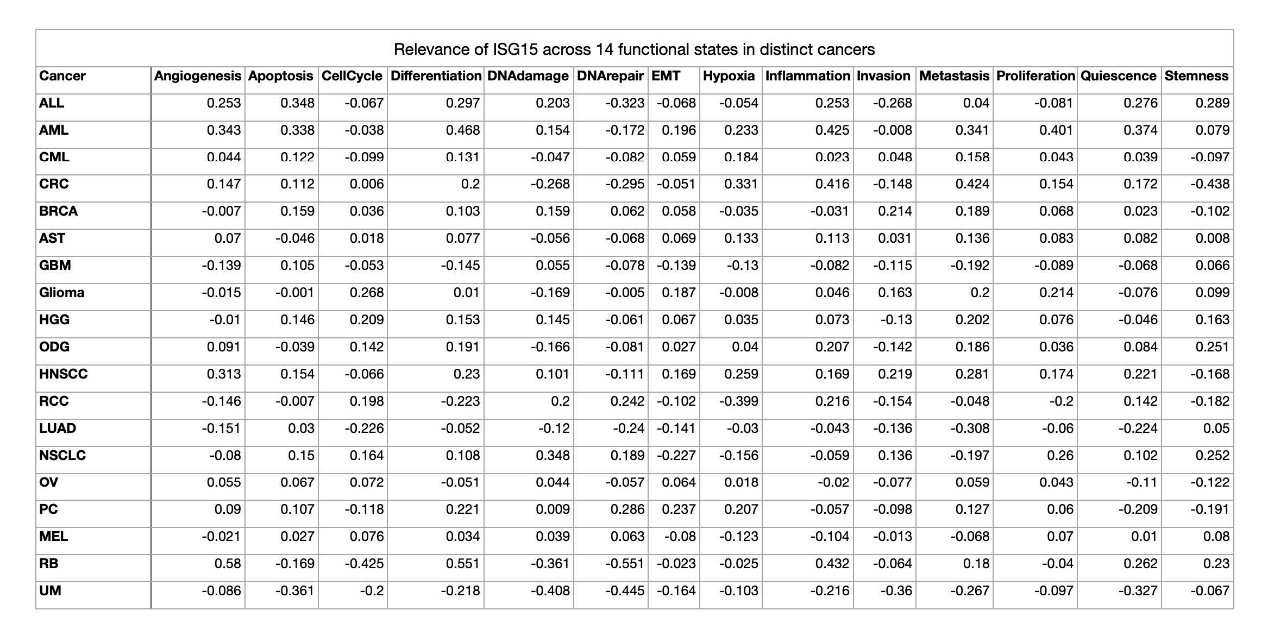

Supplement: Supplementary file 1 — Supplementary file1 (DOCX 2283 KB) [file 262_2025_4026_MOESM1_ESM.docx]
